# Mortality trends in diabetes with proxy-defined steatotic liver disease in the United States, 1999–2023

**DOI:** 10.3389/fendo.2026.1828354

**Published:** 2026-06-12

**Authors:** Hongzhou Liu, Ru Wang, Liqin Cui, Linlin Wang, Jia Liu, Yangfan Du, Song Dong, Kaide Xia

**Affiliations:** 1 Department of Endocrinology, Aerospace Center Hospital, Beijing, China; 2 Department of Endocrinology, Beijing Jishuitan Hospital, Beijing, China; 3 Guiyang Maternal and Child Health Care Hospital, Guiyang Children’s Hospital, Guiyang, Guizhou, China

**Keywords:** death certificates, diabetes mellitus, metabolic dysfunction–associated steatotic liver disease, mortality, steatotic liver disease

## Abstract

**Background:**

To characterize U.S. mortality trends for diabetes mellitus (DM), proxy-defined metabolic dysfunction–associated steatotic liver disease (MASLD), and deaths listing both conditions, and to present exploratory conditional overlap extrapolations through 2040.

**Methods:**

We analyzed CDC WONDER multiple-cause-of-death data from 1999–2023 among adults aged ≥25 years. DM was identified by ICD-10 codes E10–E14 and steatotic liver disease by K75.8 or K76.0. Because clinical MASLD cannot be directly ascertained in CDC WONDER, MASLD was defined using a death-certificate proxy combining steatotic liver disease codes with recorded cardiometabolic conditions. Age-adjusted mortality rates were assessed using Joinpoint regression. Prophet models generated conditional extrapolations through 2040. Sensitivity analyses used K76.0-only coding, K75.8-only coding, restriction to 1999–2019, and an underlying-cause-of-death-only analysis requiring K76.0 to be listed as the underlying cause of death.

**Results:**

DM-involved mortality changed little from 1999 to 2023 (118.47 to 122.61 per 100,000; average annual percent change [AAPC], −0.03%). In contrast, overlap mortality increased from 0.08 to 0.84 per 100,000 (AAPC, 11.20%), and proxy-defined MASLD mortality increased from 0.13 to 1.29 per 100,000 (AAPC, 9.83%), although absolute rates remained low. Sensitivity analyses using K76.0-only coding, restriction to 1999–2019, and an underlying-cause-of-death-only definition showed directionally consistent increases, whereas K75.8-only overlap deaths were sparse, zero, or suppressed. The underlying-cause-only analysis also showed a directionally consistent increase from 2000 to 2023 (AAPC, 14.46%; 95% CI, 13.46 to 21.51). Conditional extrapolations suggested continued growth through 2040 but were inherently uncertain.

**Conclusions:**

Proxy-based mortality involving co-recorded DM and steatotic liver disease increased substantially in U.S. death-certificate data. These findings were directionally consistent across alternative coding, period-restricted, and underlying-cause-only sensitivity analyses but remain limited by under-ascertainment, misclassification, and evolving death-certificate documentation. They should be interpreted as descriptive surveillance patterns rather than evidence of biological expansion or causal mechanisms.

## 1 Introduction

Diabetes mellitus (DM) remains a leading contributor to premature mortality and a major driver of cardiometabolic multimorbidity in the United States (U.S.) (1–4). In parallel, steatotic liver disease—now reframed under the umbrella of metabolic dysfunction–associated steatotic liver disease (MASLD)—has emerged as a common and clinically consequential comorbidity in populations living with metabolic risk factors (5–8). However, contemporary clinical MASLD criteria cannot be directly applied in national death-certificate data. Accordingly, mortality surveillance studies must rely on proxy definitions based on available ICD-10 coding and co-recorded metabolic conditions. The co-occurrence of DM and steatotic liver disease, hereafter termed diabetes mellitus with steatotic liver disease (DMSLD), is associated with higher risks of cardiovascular events, progressive liver disease, and competing causes of death, yet population-level mortality patterns capturing this overlap remain incompletely characterized (6, 7).

Existing national trend studies using death certificate data have typically examined DM or individual diabetes-related complications in isolation, with limited attention to the evolving relationship between DM and steatotic liver disease over time (3, 9). Moreover, the COVID-19 era introduced substantial perturbations to chronic disease mortality, raising the question of whether observed overlap trends reflect longer-term structural changes or short-term shocks (10). Finally, understanding what people die from within overlap populations—via underlying cause-of-death (UCOD) composition—can clarify whether overlap mortality is dominated by cardiometabolic causes, liver-related causes, or a shifting mixture of multiple domains (8, 11).

Using the CDC WONDER Multiple Cause of Death database (1999–2023), we quantified national mortality burden and long-term trends for DM, proxy-defined MASLD, and their overlap, DMSLD. We further characterized overlap dynamics using two relationship metrics (penetration and contribution), evaluated temporal inflection points and COVID-era deviations, generated conditional extrapolations through 2040, and examined how underlying causes of death shifted over time and by sex. Throughout, MASLD-related findings are intended to represent death-certificate–based proxy phenotypes rather than clinically adjudicated MASLD.

## 2 Materials and methods

### 2.1 Data source and study population

We used the U.S. Centers for Disease Control and Prevention Wide-ranging Online Data for Epidemiologic Research (CDC WONDER) Multiple Cause of Death database to identify deaths occurring from 1999 through 2023 among adults aged ≥25 years (12–14). CDC WONDER provides national death certificate data, including underlying cause of death (UCOD) and multiple cause of death (MCOD) coded using the International Classification of Diseases, 10th Revision (ICD-10). This study was deemed exempt from institutional review board review because it used deidentified, publicly available government data. No individual-level identifiers were accessed. Records suppressed by CDC WONDER for confidentiality were excluded from analysis.

### 2.2 Study design

Deaths involving diabetes mellitus (DM) were identified when ICD-10 codes E10–E14 were listed anywhere on the death certificate (multiple-cause-of-death fields). Steatotic liver disease (SLD) was identified using ICD-10 codes K75.8 or K76.0. Because contemporary clinical MASLD cannot be directly ascertained in CDC WONDER, deaths involving MASLD were operationalized using a death-certificate proxy adapted to the constraints of available ICD-10 coding. Specifically, proxy-defined MASLD included decedents with SLD (K75.8 or K76.0) and at least one cardiometabolic risk factor recorded on the death certificate, including diabetes (E10–E14), hypertension (I10), dyslipidemia (E78), or obesity (E66), regardless of whether these conditions were listed as the underlying or contributing causes. This operational definition was intended to approximate a metabolic steatotic liver disease phenotype in mortality surveillance data; however, it does not fully reproduce contemporary clinical MASLD criteria, cannot fully exclude alternative liver etiologies, and should not be interpreted as a clinically adjudicated MASLD diagnosis. Overlap deaths, termed diabetes mellitus with steatotic liver disease, were defined as deaths with both DM and SLD recorded on the same death certificate (15).

We obtained annual death counts and age-adjusted mortality rates (AAMR) per 100,000 persons directly from CDC WONDER query outputs, standardized to the 2000 U.S. standard population, along with 95% confidence intervals (CIs). Analyses were stratified by age group (25–64 and ≥65 years), sex (male and female), race (White, Black, and Other), U.S. Census region (Midwest, Northeast, South, and West), and urbanization (metropolitan vs nonmetropolitan), using the classifications available in CDC WONDER.

To quantify the evolving relationship between DM and steatotic liver disease mortality, we calculated two standardized rate ratios: SRR1 (penetration), defined as AAMR_DMSLD_/AAMR_DM_, and SRR2 (contribution), defined as AAMR_DMSLD_/AAMR_MASLD_. SRR1 summarizes the relative position of DMSLD mortality within DM-involved mortality, whereas SRR2 summarizes the relative position of diabetes–SLD overlap within proxy-defined MASLD-involved mortality. These metrics are population-level rate relationship indicators and should not be interpreted as individual risk measures, conditional probabilities, or causal effect estimates. All MASLD-related analyses should therefore be interpreted as analyses of proxy-defined death-certificate phenotypes rather than clinically confirmed disease entities.

### 2.3 Statistical analysis

We determined the annual percent change (APC) and the average annual percentage change (AAPC) with 95% CIs in AAMR using the Joinpoint Regression Program (Joinpoint version 5.4.0.0, National Cancer Institute), which fits log-linear regression models to identify temporal segments connected by joinpoints (16). We used Prophet time-series models to (1) estimate short-term counterfactual trajectories during the COVID-19 era (2020–2022) based on pre-pandemic trends (1999–2019) and quantify deviations between observed and expected values, and (2) generate conditional extrapolations of AAMR and SRR trajectories through 2040 with uncertainty intervals (17). These long-term model outputs were intended as historical-trend extrapolations rather than scenario-based forecasts and should be interpreted accordingly. Models were fit to annual series overall and by key subgroups, consistent with the presentation in figures. Among overlap deaths, we summarized UCOD composition across periods using two complementary approaches: (1) the top 10 UCOD categories plus “other,” and (2) aggregation of UCOD into broader cause domains (e.g., cardiovascular disease, endocrine/metabolic causes, liver-related causes, cancers). For period-based UCOD analyses, calendar years were grouped as 1999–2005, 2006–2010, 2011–2015, 2016–2019, and 2020–2023. We further examined sex-stratified UCOD compositions to assess heterogeneity in cause structure. To evaluate the robustness of the DMSLD mortality trends to coding assumptions, underlying-cause attribution, and pandemic-era deviations, we performed four sensitivity analyses feasible within the CDC WONDER aggregate query interface. In S1, we repeated the DMSLD analysis using K76.0 only, a more specific fatty liver code. In S2, we repeated the analysis using K75.8 only to assess whether the primary trend was driven by this broader code. In S3, we repeated the primary DMSLD trend analysis after restricting the study period to 1999–2019 to assess whether the observed increase was driven primarily by pandemic-era deviations or recent documentation changes. In S4, we performed an underlying-cause-of-death-only sensitivity analysis in which K76.0 was required to be listed as the underlying cause of death, while diabetes codes E10–E14 were identified anywhere on the death certificate. This analysis was designed to assess whether the primary multiple-cause DMSLD trend was driven solely by increased documentation of K76.0 as a contributing cause of death. Because CDC WONDER aggregate outputs do not allow simultaneous implementation of multiple inclusion criteria together with exclusion of competing liver etiologies while preserving directly computed age-adjusted mortality rates and confidence intervals, we did not derive exclusion-based age-adjusted rates by subtraction. Data processing and analysis were performed using R (version 4.5.0), along with Zstats v1.0 (www.zstats.net). A two-sided P-value < 0.05 was considered statistically significant.

## 3 Results

### 3.1 Overall burden and long-term trends of DM, DMSLD, and MASLD

**Figure 1:**
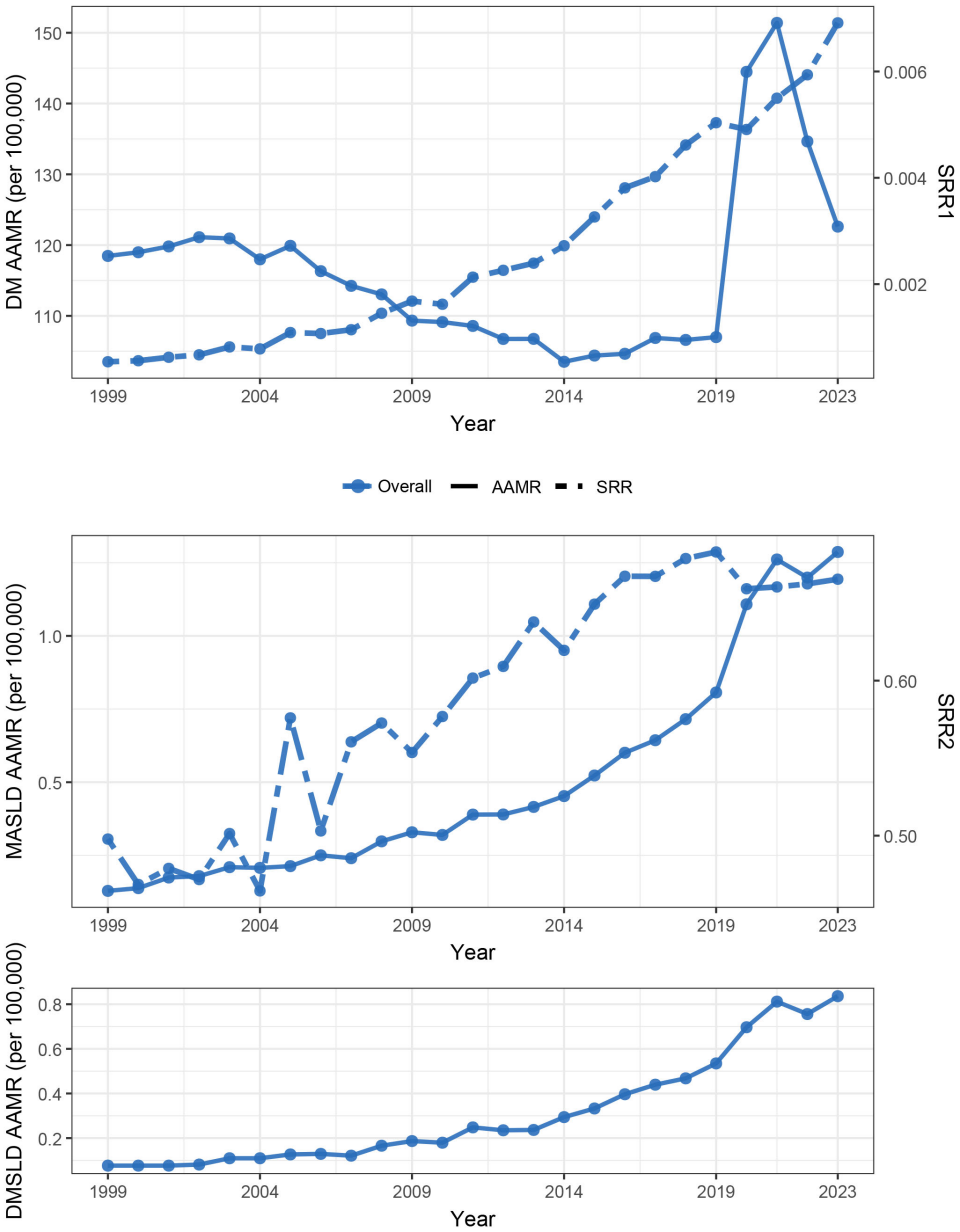
Trends in DM, proxy-defined MASLD, and DMSLD AAMR and overlap metrics (SRR1 and SRR2). Abbreviations: AAMR, age-adjusted mortality rate; DM, diabetes mellitus; DMSLD, diabetes mellitus with steatotic liver disease; MASLD, proxy-defined metabolic dysfunction–associated steatotic liver disease; SRR, standardized rate ratio; SRR1, penetration (AAMR_DMSLD_/AAMR_DM_); SRR2, contribution (AAMR_DMSLD_/AAMR_MASLD_).

Between 1999 and 2023, DM-involved deaths increased from 209,354 to 338,877, whereas the AAMR changed modestly from 118.47 to 122.61 per 100,000, indicating minimal net change over the study period (AAPC −0.03%; 95% CI, −0.39 to 0.27). In comparison, DMSLD deaths increased from 113 to 2,343 and the AAMR increased from 0.08 to 0.84 per 100,000 (AAPC 11.20%), and proxy-defined MASLD deaths increased from 227 to 3,521 with the AAMR increasing from 0.13 to 1.29 per 100,000 (AAPC 9.83%). Thus, both DMSLD and proxy-defined MASLD AAMRs increased markedly in relative terms over the 25-year period (**Table 1**; **Figure 1**), although the absolute AAMRs for both outcomes remained low throughout the study period.

**Table 1:** Overall burden and temporal trends for DM, DMSLD, and MASLD.

Characteristics	1999		2023		AAPC (95% CI)
	Deaths (N)	AAMR, per 100,000 persons	Deaths (N)	AAMR, per 100,000 persons	
DM	209354	118.47 (117.96, 118.97)	338877	122.61 (122.19, 123.03)	-0.03 (-0.39 to 0.27)
DMSLD	113	0.08 (0.06, 0.09)	2343	0.84 (0.80, 0.87)	11.2 (10.47 to 12.11)
MASLD	227	0.13 (0.11, 0.15)	3521	1.29 (1.24, 1.33)	9.83 (9.41 to 10.39)

MASLD indicates proxy-defined metabolic dysfunction–associated steatotic liver disease in this study. Abbreviations: DM, diabetes mellitus; DMSLD, diabetes mellitus with steatotic liver disease; MASLD, metabolic dysfunction–associated steatotic liver disease; AAMR, age-adjusted mortality rate; AAPC, average annual percent change; CI, confidence interval.

Joinpoint regression analysis identified distinct temporal segments superimposed on these long-term patterns. The AAMR for DM declined during 1999–2018 (APC −0.92%), increased during 2018–2021 (APC 14.48%), and declined during 2021–2023 (APC −11.15%). Against a background of long-term increases, both DMSLD and proxy-defined MASLD showed steeper increases during the pandemic-era interval. The APC for DMSLD increased from 11.02% (1999–2018) to 19.97% (2018–2021), and the APC for proxy-defined MASLD increased from 9.08% (1999–2018) to 22.48% (2018–2021). During 2021–2023, neither DMSLD nor proxy-defined MASLD changed significantly (DMSLD: APC 0.73%; proxy-defined MASLD: APC −0.43%; both non-significant) (**Supplementary Table 1**). Given the low absolute rates and the limitations of death-certificate ascertainment, these changes should be interpreted cautiously.

In subgroup analyses, DM AAMR increases were more pronounced among adults aged 25–64 years (AAPC 0.62%), residents in the South (AAPC 0.41%), males, and nonmetropolitan areas (AAPC 1.30%) (**Supplementary Tables 1**, **2**). Conversely, DMSLD and proxy-defined MASLD increases were steeper among individuals aged ≥65 years and those in nonmetropolitan areas (DMSLD: AAPC 16.86% for ≥65 years; AAPC 15.39% for nonmetropolitan; MASLD: AAPC 14.75% for ≥65 years; AAPC 13.38% for nonmetropolitan) (**Supplementary Table 2**; **Supplementary Figures 1–5**).

### 3.2 Overlap relationship metrics: SRR1 and SRR2

Overall, SRR1 increased substantially over time, whereas SRR2 increased modestly and then plateaued. SRR1 increased during 1999–2017 (AAPC 11.00%) and continued to increase during 2017–2023 (APC 8.14%). The SRR1 increase was larger among individuals aged ≥65 years (AAPC 16.64%), nonmetropolitan (AAPC 13.81%), and females (AAPC 12.66%). In contrast, SRR2 increased slightly during 1999–2017 (AAPC 1.16%) and did not change significantly during 2017–2023 (APC −0.66%; 95% CI includes 0) (**Supplementary Table 3**; **Supplementary Figure 6**).

### 3.3 Sensitivity analyses

In sensitivity analyses, the increasing DMSLD mortality trend was directionally consistent under alternative coding, period-restricted, and underlying-cause assumptions (**Supplementary Table 4**). In S1, when DMSLD was restricted to K76.0-coded fatty liver, deaths increased from 104 in 1999 to 2,281 in 2023, and the AAMR increased from 0.08 to 0.82 per 100,000, with an AAPC of 10.77% (95% CI, 9.99 to 11.78). In S2, the K75.8-only DMSLD definition yielded sparse, zero, or suppressed annual counts and was therefore not suitable for Joinpoint trend modeling, indicating that the primary DMSLD trend was not driven by the broader K75.8 code. In S3, when the primary DMSLD analysis was restricted to the pre-pandemic period of 1999–2019, deaths increased from 113 to 1,422 and AAMR increased from 0.08 to 0.53 per 100,000, with an AAPC of 11.16% (95% CI, 10.54 to 12.14). In S4, requiring K76.0 to be listed as the underlying cause of death yielded a directionally consistent increase: deaths rose from 24 in 2000 to 940 in 2023, and AAMR increased from 0.01 to 0.31 per 100,000, with an AAPC of 14.46% (95% CI, 13.46 to 21.51). This supports the directional consistency of the primary DMSLD trend under a more restrictive death-certificate definition and suggests that it was not solely driven by increased contributing-cause documentation, although early counts were low and residual coding effects remain possible.

### 3.4 Prophet counterfactuals and projections to 2040

**Figure 2:**
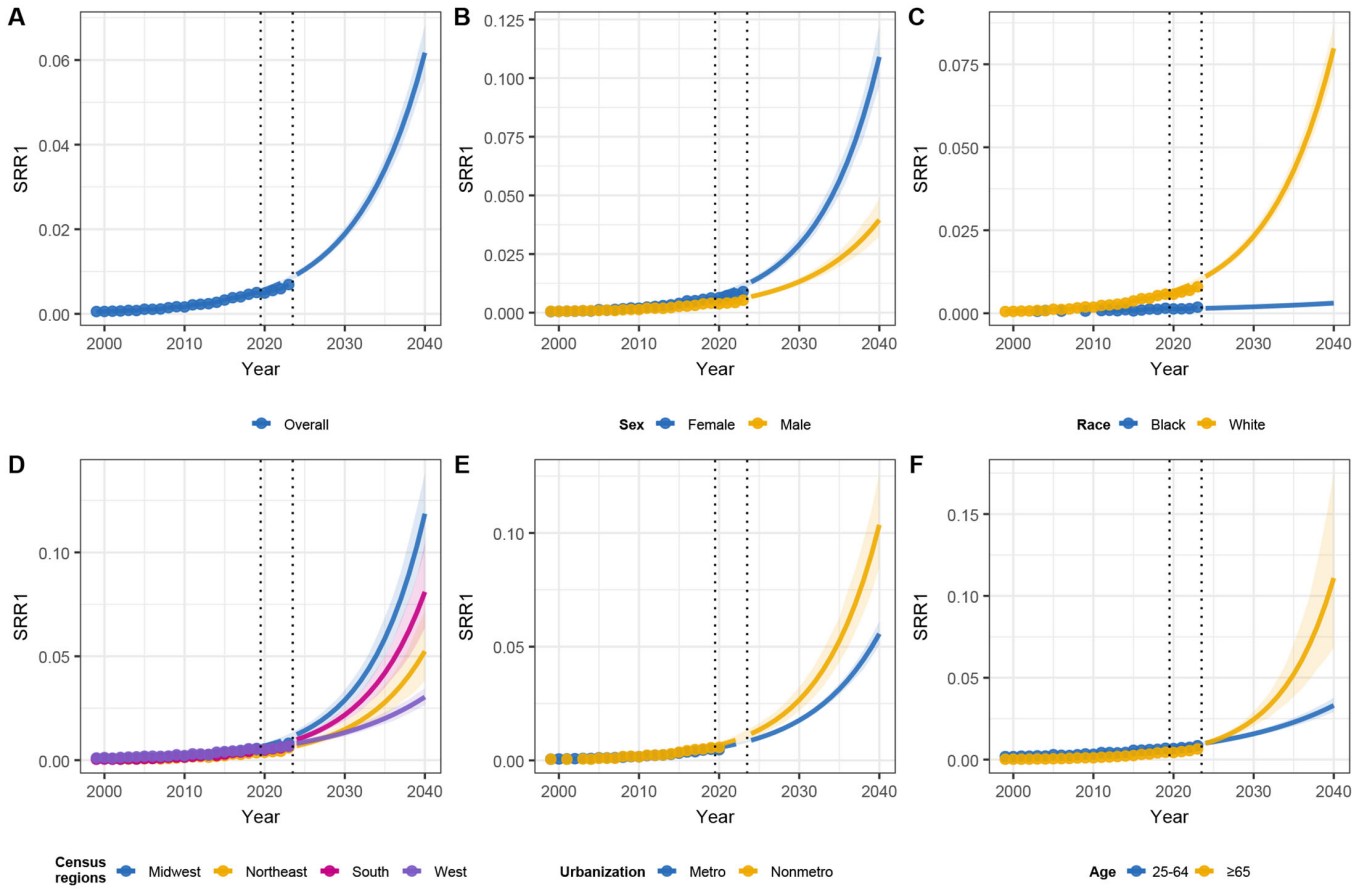
Trends in SRR1 and Prophet-based conditional extrapolations through 2040. **(A)** overall; **(B)** sex; **(C)** race; **(D)** census region; **(E)** urbanization; **(F)** age (training: 1999–2019; 2020–2023 excluded). SRR1, standardized rate ratio 1 (penetration); metro, metropolitan; nonmetro, nonmetropolitan.

**Figure 3:**
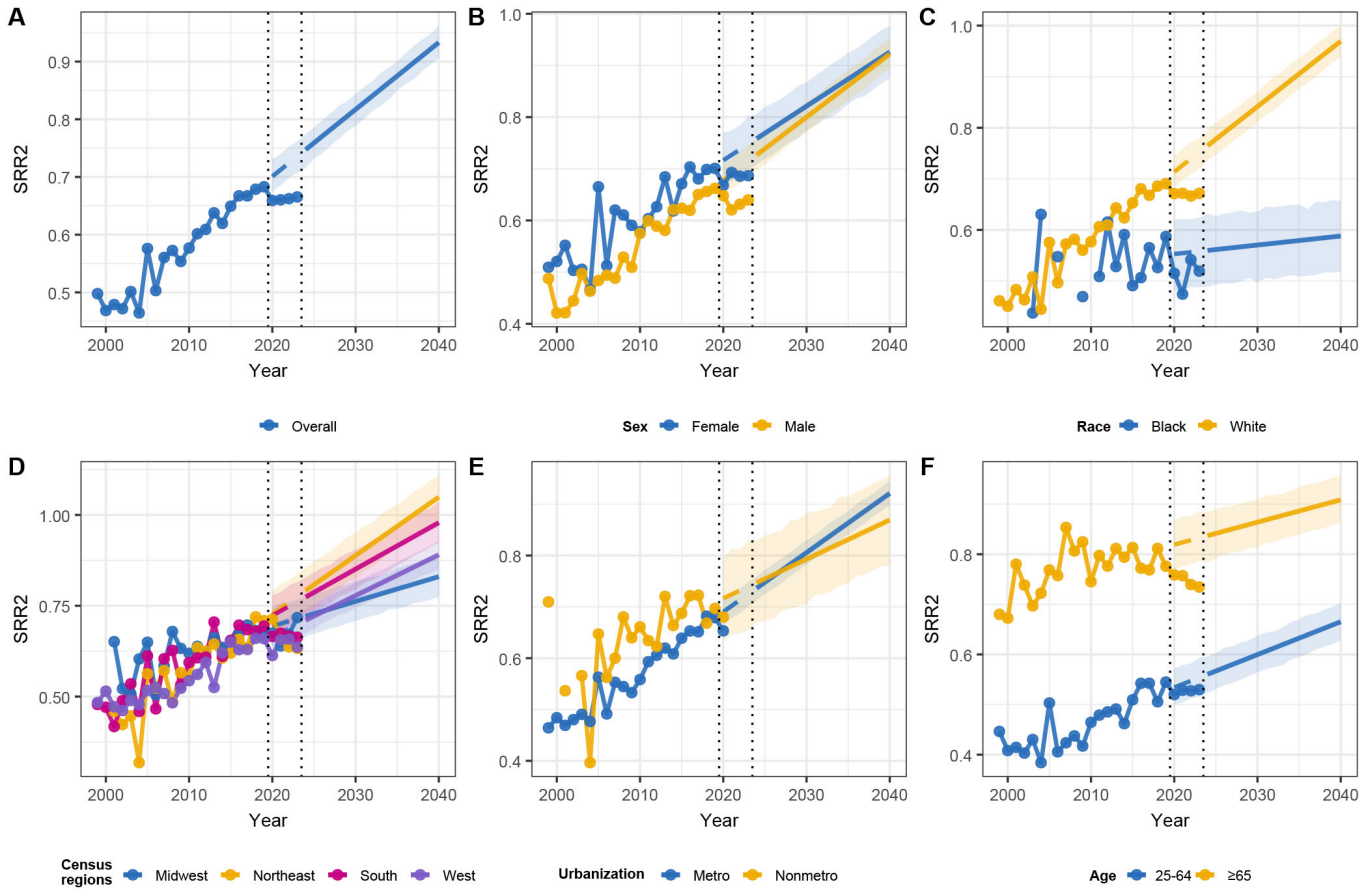
Trends in SRR2 and Prophet-based conditional extrapolations through 2040. **(A)** overall; **(B)** sex; **(C)** race; **(D)** census region; **(E)** urbanization; **(F)** age (training: 1999–2019; 2020–2023 excluded). SRR2, standardized rate ratio 2 (contribution); metro, metropolitan; nonmetro, nonmetropolitan.

Using Prophet models, we estimated counterfactual trajectories based on pre-pandemic trends and generated conditional extrapolations to 2040. Observed AAMRs for DM, DMSLD, and proxy-defined MASLD during 2020–2022 exceeded the counterfactual estimates derived from 1999–2019 trends, indicating positive deviations during the pandemic-era interval relative to pre-pandemic historical patterns. By 2023, observed values moved closer to the expected trajectories, although differences across outcomes persisted (**Supplementary Figures 7–9**). For overlap indicators, model-based extrapolations under continuation of historical trends suggested that SRR1 may continue to increase through 2040, whereas SRR2 may remain relatively stable or change slowly (**Figures 2**, **3**). These long-term estimates should be interpreted cautiously because they depend on model structure, do not incorporate alternative clinical scenarios, and assume that past patterns remain informative for future trajectories.

### 3.5 UCOD composition of overlap deaths

**Figure 4:**
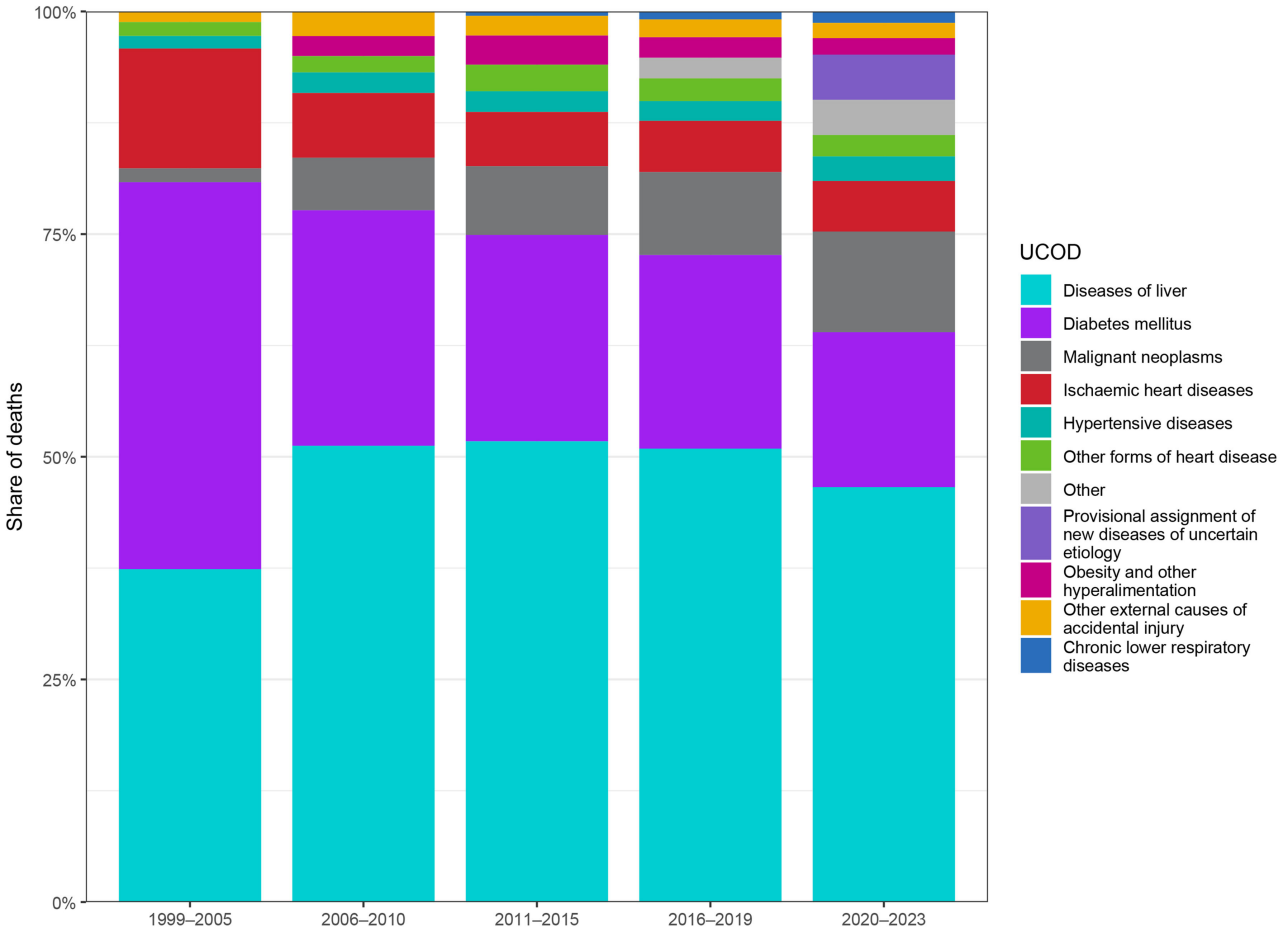
UCOD composition among DMSLD overlap deaths by period (top 10 causes and other). DMSLD, diabetes mellitus with steatotic liver disease; UCOD, underlying cause of death.

Among DMSLD overlap deaths, the leading UCOD categories across periods were dominated by liver-related causes and diabetes, with cardiovascular causes and other causes comprising major competing categories (**Figure 4**). Sex-stratified analyses showed differences in both the ranking and temporal changes of top UCOD categories (**Supplementary Figure 10**). Among MASLD-involved deaths, liver-related causes remained dominant, while cardiovascular and other causes varied over time, with discernible sex differences (**Supplementary Figures 11, 12**).

When UCODs were grouped into broader domains, DMSLD overlap deaths were primarily composed of liver-related and other domains, with the CVD-related domain contributing a smaller but consistent share and the kidney-related domain accounting for a low proportion; the liver-related domain increased over time (**Supplementary Figure 13**). Sex-stratified results demonstrated systematic differences in domain composition (**Supplementary Figure 14**). Similar domain-level patterns were observed for MASLD-involved deaths (**Supplementary Figures 15, 16**).

## 4 Discussion

In this national analysis of U.S. death certificate data from 1999–2023, we found three overarching patterns. First, DM-involved mortality remained at a persistently high level, with overall AAMR showing an extended plateau despite substantial year-to-year variation. Second, mortality involving proxy-defined steatotic liver disease coding and the DM–steatotic liver disease overlap increased markedly over the past two decades in death certificate data, although the absolute rates remained low and these patterns may reflect a combination of true epidemiologic change, evolving recognition, and coding practices. Third, relationship metrics indicated increasing overlap penetration relative to DM-involved mortality, whereas the contribution of DM within proxy-defined MASLD-involved mortality rose more modestly and appeared to plateau in more recent years. Importantly, SRR1 and SRR2 are population-level rate relationship indicators rather than individual-level risk measures, conditional probabilities, or causal parameters; they are intended to describe how the overlap pattern changes relative to the parent mortality baselines of DM and proxy-defined MASLD.

Joinpoint-segmented trends highlighted a distinct COVID-era perturbation, with a sharp increase in DM-involved AAMR during 2018–2021 followed by a subsequent decline (18). In contrast, the overlap phenotypes (DMSLD and proxy-defined MASLD) exhibited long-term increases with steeper growth around the pandemic-era interval and attenuation thereafter (19). Prophet-based counterfactual modeling showed that observed values exceeded expected trajectories during 2020–2022; however, because the absolute rates were low, no formal interrupted time-series design was applied, and the analysis was based on death-certificate proxy phenotypes, these deviations should be interpreted as suggestive temporal patterns rather than definitive evidence of a true biological acceleration or a causal pandemic effect (20). More broadly, the observed increases may reflect a combination of changing disease occurrence, heightened recognition, and evolving coding practices (21).

The expanded sensitivity analyses provide additional support for the directional consistency of the observed DMSLD trend. The K76.0-only analysis showed an increase similar to the primary DMSLD definition, whereas the K75.8-only analysis yielded sparse or suppressed counts and was not suitable for reliable trend modeling. This suggests that the primary DMSLD trend was unlikely to be driven primarily by the broader K75.8 code. In addition, the increasing trend persisted when the analysis was restricted to 1999–2019, suggesting that the observed rise was not solely attributable to pandemic-era deviations. Importantly, the newly added underlying-cause-of-death-only analysis demonstrated a directionally consistent increase when K76.0 was required to be recorded as the underlying cause of death. This analysis more directly addresses the possibility that the primary multiple-cause findings were driven solely by increased documentation of fatty liver as a contributing condition. Nevertheless, these analyses remain coding-robustness checks rather than clinical validation studies, and residual under-ascertainment, etiologic misclassification, and temporal changes in death-certificate documentation cannot be excluded.

We observed that the increase in overlap burden and SRR1 was most pronounced among older adults, nonmetropolitan populations, and females. These subgroup differences should be interpreted cautiously because this dataset does not contain clinical information on fibrosis stage, liver disease severity, BMI, glycemic control, duration of diabetes, treatment exposure, or care access, and therefore cannot test specific mechanisms. The observed patterns may reflect a combination of true epidemiologic change, differential recognition of steatotic liver disease, and evolving death-certification practices (22). Although prior literature supports the clinical relevance of liver risk assessment in people with diabetes (23), our subgroup findings should be interpreted as surveillance signals rather than direct evidence of the causal pathways underlying these disparities.

The lack of clinical granularity also limits the actionable utility of these surveillance-level patterns. CDC WONDER does not include fibrosis stage, histologic confirmation, body mass index, glycemic control, diabetes duration, alcohol exposure, medication use, treatment history, healthcare access, or longitudinal clinical trajectories. Therefore, the present analysis cannot identify which patients are at highest risk, determine whether mortality increases are driven by advanced fibrosis versus cardiometabolic comorbidity, or inform specific screening, referral, monitoring, or treatment thresholds. The observed subgroup differences should not be interpreted as evidence supporting patient-level risk stratification or specific clinical decision-making. Rather, they should be viewed as population-level signals that can help prioritize future validation studies using clinically enriched cohorts, registry-based datasets, claims-linked data, or electronic health record–based analyses.

Beyond rate trends, UCOD analyses suggested that overlap mortality is characterized by a multi-domain cause structure rather than a single dominant pathway (24, 25). The re-ranking of top UCOD categories over time and the domain-level composition shifts are consistent with a complex multimorbidity profile in which cardiometabolic, liver-related, and other competing causes co-occur within mortality data (24, 26). Sex-stratified UCOD compositions further indicate heterogeneity in cause structure, although the dataset does not permit direct assessment of the clinical pathways leading to death (27, 28). Clinically, these findings support integrated rather than siloed risk assessment, but they should be interpreted as descriptive mortality patterns rather than mechanistic evidence regarding terminal disease processes (26).

From a clinical practice perspective, the sustained rise in overlap penetration within DM mortality is consistent with the need to maintain attention to identification of steatotic liver disease in people with diabetes, particularly in older adults and populations facing access barriers (24, 29, 30). Although liver risk stratification in people with diabetes is supported by prior guidelines (31), the present study does not allow inference regarding the effect of any specific clinical strategy on mortality outcomes. Nor can it determine which individuals should undergo intensified screening, referral, monitoring, or treatment on the basis of the observed mortality patterns alone. These findings should therefore be interpreted as descriptive surveillance signals that require validation in clinically characterized datasets before being translated into patient-level decision-making. Likewise, population-level prevention strategies targeting obesity, metabolic risk factors, and access to coordinated care are relevant from a public health perspective (30, 32), although their specific contribution to the trends observed here cannot be established with this dataset.

Strengths of this study include national coverage, long-term follow-up across the ICD-10 era, standardized AAMR estimation, complementary trend methods, and the introduction of relationship metrics that capture overlap dynamics beyond absolute rates. Several limitations warrant consideration. The most important limitation, and one central to the validity of our findings, is that contemporary clinical MASLD could not be directly identified in CDC WONDER and was instead approximated using a death-certificate proxy. Because liver disease is incompletely captured on death certificates and the available ICD-10 codes lack etiologic specificity, both under-ascertainment and misclassification are likely. In addition, because DMSLD was defined by co-listing of DM and SLD on the death certificate, apparent increases over time may partly reflect improved recognition and reporting of SLD rather than only true changes in underlying disease occurrence. This distinction is especially important because the absolute mortality rates for DMSLD and proxy-defined MASLD remained low throughout the study period. The underlying-cause-of-death-only sensitivity analysis reduces, but does not eliminate, concern that the primary multiple-cause findings were driven by increased contributing-cause documentation, residual misclassification, under-ascertainment, or evolving diagnostic recognition and coding practices. The dataset also lacks fibrosis stage, histologic confirmation, liver disease severity, BMI, glycemic control, duration of diabetes, alcohol exposure, medication use, treatment history, healthcare access, and other individual-level clinical or care-related variables. Accordingly, mechanistic attribution and causal inference are not possible, subgroup differences should be interpreted as descriptive surveillance patterns rather than direct evidence of specific biological or healthcare-system drivers, and the findings cannot support patient-level risk stratification, screening thresholds, referral decisions, or treatment recommendations. In addition, the Prophet-based long-term estimates are conditional extrapolations rather than scenario-based forecasts. They assume that historical patterns remain informative and do not explicitly account for future changes in screening, coding, treatment uptake, or the effects of emerging therapies for diabetes and MASLD. In particular, because CDC WONDER aggregate outputs did not allow exclusion-based restricted definitions while preserving directly calculated AAMRs, alternative liver etiologies could not be fully excluded. Therefore, the sensitivity analyses should be viewed as coding-robustness checks rather than validation of the MASLD proxy definition. In addition, no alternative scenario-based projections were modeled; therefore, the long-term extrapolations remain inherently uncertain and should be interpreted cautiously. Future work linking mortality surveillance with clinically characterized datasets, incorporating alternative modeling strategies, or conducting sensitivity analyses using alternative SLD coding strategies could help clarify the balance between true burden change and documentation effects.

## 5 Conclusions

Proxy-based mortality involving co-recorded diabetes and steatotic liver disease increased substantially in U.S. death certificate data from 1999 to 2023, with increasing overlap penetration relative to DM-involved mortality. Sensitivity analyses using K76.0-only coding, restriction to the pre-pandemic period, and an underlying-cause-of-death-only strategy supported the directional consistency of this observed increase within the constraints of CDC WONDER, suggesting that the trend was not solely driven by inclusion of the broader K75.8 code, pandemic-era deviations, or increased documentation of K76.0 as a contributing cause of death. However, the absolute mortality rates remained low, and these sensitivity analyses do not eliminate the possibility of residual misclassification, under-ascertainment, or evolving death-certificate documentation. Because the dataset lacks clinical information on disease severity, metabolic control, treatment exposure, alcohol exposure, healthcare access, longitudinal clinical trajectories, and other individual-level factors, the mechanisms underlying these patterns cannot be established. The observed subgroup differences and temporal changes should therefore be interpreted cautiously as descriptive surveillance findings rather than direct evidence of specific biological or structural drivers, and they cannot directly inform patient-level risk stratification, screening thresholds, referral decisions, or treatment recommendations. Long-term model outputs should likewise be interpreted as conditional extrapolations under continuation of historical patterns rather than precise forecasts. Within these constraints, these findings may help inform future epidemiologic and clinical studies, but any practice implications require validation in clinically characterized datasets.

## Data Availability

Publicly available datasets were analyzed in this study. These data can be found in the CDC WONDER Multiple Cause of Death database: https://wonder.cdc.gov/mcd-icd10.html and https://wonder.cdc.gov/mcd-icd10-expanded.html. The processed results supporting the conclusions of this article are included in the article/**Supplementary Material**.
