# Supplementary material for: Mortality trends in diabetes with proxy-defined steatotic liver disease in the United States, 1999–2023
Source: Front Endocrinol (Lausanne). 2026 Jun 12;17:1828354. doi: 10.3389/fendo.2026.1828354 (PMC13303214; doi:10.3389/fendo.2026.1828354)
Supplement: Supplementary file 1 [file DataSheet1.docx]

**Supplementary material:**

**Mortality trends in diabetes with proxy-defined steatotic liver disease in the United States, 1999–2023**

**Content**

[**Supplementary tables： 3**](#_Toc230357061)

[**Table S1. Joinpoint-segmented APC in AAMR for DM, DMSLD, and MASLD 3**](#_Toc230357062)

[**Table S2. Burden and temporal trends in DM, DMSLD and MASLD mortality stratified by characteristics 6**](#_Toc230357063)

[**Table S3. Joinpoint-estimated APC and AAPC in SRR1 and SRR2 8**](#_Toc230357064)

[**Table S4. Sensitivity analyses of DMSLD mortality trends under alternative coding, period, and underlying-cause assumptions 10**](#_Toc230357065)

[**Supplementary figures： 11**](#_Toc230357066)

[**Figure S1. Trends in DM, MASLD, and DMSLD AAMR and overlap metrics (SRR1 and SRR2) by sex. 11**](#_Toc230357067)

[**Figure S2. Trends in DM, MASLD, and DMSLD AAMR and overlap metrics (SRR1 and SRR2) by race. 12**](#_Toc230357068)

[**Figure S3. Trends in DM, MASLD, and DMSLD AAMR and overlap metrics (SRR1 and SRR2) by census regions. 13**](#_Toc230357069)

[**Figure S4. Trends in DM, MASLD, and DMSLD AAMR and overlap metrics (SRR1 and SRR2) by urbanization. 14**](#_Toc230357070)

[**Figure S5. Trends in DM, MASLD, and DMSLD AAMR and overlap metrics (SRR1 and SRR2) by age. 15**](#_Toc230357071)

[**Figure S6. Joinpoint-identified trends in SRR1 (penetration) and SRR2 (contribution) by subgroup. 16**](#_Toc230357072)

[**Figure S7. Trends in DM AAMR and Prophet-based forecasts through 2040. 17**](#_Toc230357073)

[**Figure S8. Trends in DMSLD AAMR and Prophet-based forecasts through 2040. 18**](#_Toc230357074)

[**Figure S9. Trends in MASLD AAMR and Prophet-based forecasts through 2040. 19**](#_Toc230357075)

[**Figure S10. UCOD composition among DMSLD overlap deaths (top 10 causes and other) by sex. 20**](#_Toc230357076)

[**Figure S11. UCOD composition among MASLD overlap deaths by period (top 10 causes and other). 21**](#_Toc230357077)

[**Figure S12. UCOD composition among MASLD overlap deaths (top 10 causes and other) by sex. 22**](#_Toc230357078)

[**Figure S13. UCOD domain composition among DMSLD overlap deaths. 23**](#_Toc230357079)

[**Figure S14. UCOD domain composition among DMSLD overlap deaths by sex. 24**](#_Toc230357080)

[**Figure S15. UCOD domain composition among MASLD overlap deaths. 25**](#_Toc230357081)

[**Figure S16. UCOD domain composition among MASLD overlap deaths by sex. 26**](#_Toc230357082)

# **Supplementary tables：**

| **Table S1. Joinpoint-segmented APC in AAMR for DM, DMSLD, and MASLD** | | | | | | | |  |
| --- | --- | --- | --- | --- | --- | --- | --- | --- |
| **Characteristics** | | **Segment** | | **APC (95% CI)** | | | **P** | |
| **DM** | |  | |  | | |  | |
| Overall | | 1999-2018 | | -0.92 (-1.27 to -0.62) | | | <0.001 | |
| Overall | | 2018-2021 | | 14.48 (10.22 to 16.89) | | | <0.001 | |
| Overall | | 2021-2023 | | -11.15 (-15.45 to -6.93) | | | <0.001 | |
| **Age, y** | |  | |  | | |  | |
| 25-64 | | 1999-2018 | | -0.16 (-0.56 to 0.19) | | | 0.355 | |
| 25-64 | | 2018-2021 | | 18.66 (13.82 to 21.31) | | | <0.001 | |
| 25-64 | | 2021-2023 | | -15.34 (-20.19 to -10.89) | | | <0.001 | |
| ≥65 | | 1999-2018 | | -1.13 (-1.47 to -0.85) | | | <0.001 | |
| ≥65 | | 2018-2021 | | 13.13 (9.03 to 15.47) | | | <0.001 | |
| ≥65 | | 2021-2023 | | -9.73 (-13.79 to -5.63) | | | <0.001 | |
| **Census regions** | |  | |  | | |  | |
| Midwest | | 1999-2018 | | -0.95 (-1.3 to -0.66) | | | <0.001 | |
| Midwest | | 2018-2021 | | 12.92 (8.6 to 15.47) | | | <0.001 | |
| Midwest | | 2021-2023 | | -11.64 (-16.06 to -7) | | | <0.001 | |
| Northeast | | 1999-2017 | | -1.85 (-2.56 to -1.31) | | | 0.014 | |
| Northeast | | 2017-2020 | | 10.49 (3.19 to 13.54) | | | 0.04 | |
| Northeast | | 2020-2023 | | -5.77 (-14.52 to -0.71) | | | 0.044 | |
| South | | 1999-2018 | | -0.73 (-1.08 to -0.42) | | | <0.001 | |
| South | | 2018-2021 | | 16.65 (12.46 to 19.01) | | | <0.001 | |
| South | | 2021-2023 | | -10.65 (-14.71 to -6.78) | | | <0.001 | |
| West | | 1999-2018 | | -0.72 (-1.09 to -0.39) | | | <0.001 | |
| West | | 2018-2021 | | 13.99 (10.13 to 16.19) | | | <0.001 | |
| West | | 2021-2023 | | -11.43 (-16.04 to -7.19) | | | <0.001 | |
| **Race** | |  | |  | | |  | |
| Black | | 1999-2018 | | -1.89 (-2.47 to -1.44) | | | 0.002 | |
| Black | | 2018-2021 | | 15.89 (9.5 to 19.91) | | | 0.003 | |
| Black | | 2021-2023 | | -13.96 (-20.16 to -6.9) | | | 0.004 | |
| Other | | 1999-2018 | | -1.36 (-1.91 to -0.9) | | | <0.001 | |
| Other | | 2018-2021 | | 13.92 (8.71 to 17.49) | | | <0.001 | |
| Other | | 2021-2023 | | -15.06 (-19.71 to -9.3) | | | <0.001 | |
| White | | 1999-2018 | | -0.77 (-1.08 to -0.5) | | | <0.001 | |
| White | | 2018-2021 | | 14.23 (10.41 to 16.31) | | | <0.001 | |
| White | | 2021-2023 | | -10.22 (-14.39 to -6.41) | | | <0.001 | |
| **Sex** | |  | |  | | |  | |
| Female | | 1999-2018 | | -1.52 (-1.87 to -1.23) | | | <0.001 | |
| Female | | 2018-2021 | | 14.03 (9.79 to 16.36) | | | <0.001 | |
| Female | | 2021-2023 | | -11.29 (-15.84 to -6.98) | | | <0.001 | |
| Male | | 1999-2018 | | -0.49 (-0.84 to -0.17) | | | 0.003 | |
| Male | | 2018-2021 | | 14.63 (10.4 to 17.1) | | | <0.001 | |
| Male | | 2021-2023 | | -10.98 (-15.13 to -6.81) | | | <0.001 | |
| **Urbanization** | |  | |  | | |  | |
| Metropolitan | | 1999-2018 | | -1.13 (-1.58 to -0.75) | | | <0.001 | |
| Metropolitan | | 2018-2020 | | 16.84 (8.53 to 20.94) | | | <0.001 | |
| Nonmetropolitan | | 1999-2018 | | -0.15 (-0.45 to 0.14) | | | 0.289 | |
| Nonmetropolitan | | 2018-2020 | | 16.11 (10.2 to 19.37) | | | <0.001 | |
|  | |  | |  | | |  | |
| **DMSLD** | |  | |  | | |  | |
| Overall | | 1999-2018 | | 11.02 (8.32 to 12.26) | | | 0.013 | |
| Overall | | 2018-2021 | | 19.97 (13.77 to 23.79) | | | 0.001 | |
| Overall | | 2021-2023 | | 0.73 (-5.3 to 9.32) | | | 0.651 | |
| **Age, y** | |  | |  | | |  | |
| 25-64 | | 1999-2007 | | 0.83 (-25.15 to 7.67) | | | 0.911 | |
| 25-64 | | 2007-2023 | | 9.39 (7.91 to 16.83) | | | 0.007 | |
| ≥65 | | 1999-2005 | | 24.38 (4.41 to 113.09) | | | 0.042 | |
| ≥65 | | 2005-2021 | | 15.39 (14.46 to 44.52) | | | <0.001 | |
| ≥65 | | 2021-2023 | | 7.24 (0.88 to 14.68) | | | 0.034 | |
| **Census regions** | |  | |  | | |  | |
| Midwest | | 2001-2021 | | 14.03 (13.22 to 74.77) | | | 0.004 | |
| Midwest | | 2021-2023 | | 4.07 (-4.03 to 13.58) | | | 0.152 | |
| Northeast | | 2001-2023 | | 12.19 (10.94 to 14.44) | | | <0.001 | |
| South | | 1999-2018 | | 13.02 (6.17 to 19.55) | | | 0.029 | |
| South | | 2018-2021 | | 22.52 (15.18 to 27.87) | | | <0.001 | |
| South | | 2021-2023 | | 0.01 (-7.64 to 10.88) | | | 0.84 | |
| West | | 1999-2023 | | 9.48 (8.73 to 10.59) | | | <0.001 | |
| **Race** | |  | |  | | |  | |
| Black | | 2003-2012 | | -0.28 (-29.27 to 9.18) | | | 0.926 | |
| Black | | 2012-2023 | | 11.48 (-5.16 to 35.02) | | | 0.056 | |
| Other | | 2011-2023 | | 8.71 (5.38 to 14.16) | | | <0.001 | |
| White | | 1999-2018 | | 12.07 (7.27 to 13.56) | | | 0.02 | |
| White | | 2018-2021 | | 19.95 (13.86 to 23.92) | | | <0.001 | |
| White | | 2021-2023 | | 0.46 (-5.93 to 9.33) | | | 0.725 | |
| **Sex** | |  | |  | | |  | |
| Female | | 1999-2021 | | 13.33 (12.27 to 39.65) | | | 0.019 | |
| Female | | 2021-2023 | | 3.79 (-3.46 to 13.02) | | | 0.208 | |
| Male | | 1999-2014 | | 9.32 (7.34 to 10.88) | | | 0.001 | |
| Male | | 2014-2021 | | 16.31 (14.55 to 22.02) | | | 0.002 | |
| Male | | 2021-2023 | | 2 (-2.85 to 8.78) | | | 0.348 | |
| **Urbanization** | |  | |  | | |  | |
| Metropolitan | | 1999-2013 | | 9.02 (5.74 to 10.65) | | | 0.012 | |
| Metropolitan | | 2013-2020 | | 14.03 (12.22 to 19.31) | | | <0.001 | |
| Nonmetropolitan | | 1999-2020 | | 15.39 (14.24 to 17.47) | | | <0.001 | |
|  | |  | |  | | |  | |
| **MASLD** | |  | |  | | |  | |
| Overall | | 1999-2018 | | 9.08 (8.32 to 9.81) | | | <0.001 | |
| Overall | | 2018-2021 | | 22.48 (17.9 to 25.53) | | | <0.001 | |
| Overall | | 2021-2023 | | -0.43 (-4.94 to 4.84) | | | 0.914 | |
| **Age, y** | |  | |  | | |  | |
| 25-64 | | 1999-2018 | | 5.47 (4.38 to 6.41) | | | <0.001 | |
| 25-64 | | 2018-2021 | | 22.64 (15.33 to 27.16) | | | 0.002 | |
| 25-64 | | 2021-2023 | | -8.91 (-16.7 to 0.47) | | | 0.055 | |
| ≥65 | | 1999-2018 | | 14.78 (11.54 to 15.78) | | | 0.009 | |
| ≥65 | | 2018-2021 | | 20.28 (16.25 to 22.98) | | | <0.001 | |
| ≥65 | | 2021-2023 | | 6.68 (2.71 to 11.94) | | | 0.001 | |
| **Census regions** | |  | |  | | |  | |
| Midwest | | 1999-2021 | | 14.1 (13.52 to 21.2) | | | <0.001 | |
| Midwest | | 2021-2023 | | -0.4 (-9.24 to 12.23) | | | 0.850 | |
| Northeast | | 1999-2013 | | 5.8 (-7.56 to 8.91) | | | 0.158 | |
| Northeast | | 2013-2023 | | 12.51 (10.03 to 21.64) | | | <0.001 | |
| South | | 1999-2017 | | 10.09 (7.56 to 11.9) | | | 0.004 | |
| South | | 2017-2021 | | 22.67 (16.76 to 30.21) | | | 0.004 | |
| South | | 2021-2023 | | 0.45 (-8.01 to 10.38) | | | 0.797 | |
| West | | 1999-2023 | | 7.88 (7.14 to 9.08) | | | <0.001 | |
| **Race** | |  | |  | | |  | |
| Black | | 1999-2013 | | 2.84 (-14.45 to 7.09) | | | 0.494 | |
| Black | | 2013-2023 | | 11.73 (7.85 to 27.78) | | | 0.014 | |
| Other | | 2006-2023 | | 10.41 (8.64 to 13.6) | | | <0.001 | |
| White | | 1999-2018 | | 9.69 (8.97 to 10.37) | | | <0.001 | |
| White | | 2018-2021 | | 21.32 (17.3 to 23.98) | | | <0.001 | |
| White | | 2021-2023 | | 0.99 (-3.55 to 6.08) | | | 0.507 | |
| **Sex** | |  | |  | | |  | |
| Female | | 1999-2018 | | 10.26 (9.07 to 11.18) | | | 0.001 | |
| Female | | 2018-2021 | | 20.36 (14.66 to 23.79) | | | <0.001 | |
| Female | | 2021-2023 | | 0.11 (-5.21 to 7.04) | | | 0.854 | |
| Male | | 1999-2016 | | 8.16 (6.7 to 9.34) | | | 0.001 | |
| Male | | 2016-2021 | | 17.46 (14.52 to 24.41) | | | 0.001 | |
| Male | | 2021-2023 | | 0.69 (-5.28 to 7.62) | | | 0.711 | |
| **Urbanization** | |  | |  | | |  | |
| Metropolitan | | 1999-2018 | | 8.52 (7.22 to 9.45) | | | 0.003 | |
| Metropolitan | | 2018-2020 | | 22.89 (11.97 to 28.61) | | | <0.001 | |
| Nonmetropolitan | | 1999-2016 | | 12.2 (2.07 to 22.43) | | | 0.037 | |
| Nonmetropolitan | | 2016-2020 | | 18.55 (13.62 to 27.9) | | | <0.001 | |
| **Abbreviations:** APC, annual percent change; AAMR, age-adjusted mortality rate; DM, diabetes mellitus; DMSLD, diabetes mellitus with steatotic liver disease; MASLD, metabolic dysfunction–associated steatotic liver disease. | | | | | | | | |
| **Table S2. Burden and temporal trends in DM, DMSLD and MASLD mortality stratified by characteristics** | | | | | | | |  |
| **Characteristics** | **1999** | |  | **2023** | | **AAPC (95% CI)** | |  |
|  | **Deaths (N)** | **AAMR, per 100 000 persons** |  | **Deaths (N)** | **AAMR, per 100 000 persons** |  |  |  |
| **DM** |  |  |  |  |  |  | |  |
| **Age, y** |  |  |  |  |  |  | |  |
| 25-64 | 43505 | 30.63 (30.34, 30.92) |  | 74318 | 36.29 (36.02, 36.55) | 0.62 (0.19 to 0.94) | |  |
| ≥65 | 165849 | 480.04 (477.73, 482.36) |  | 264559 | 477.99 (476.16, 479.82) | -0.21 (-0.55 to 0.07) | |  |
| **Census regions** |  |  |  |  |  |  | |  |
| Midwest | 52975 | 125.69 (124.62, 126.76) |  | 70568 | 121.72 (120.81, 122.64) | -0.27 (-0.64 to 0.05) | |  |
| Northeast | 41931 | 112.58 (111.50, 113.65) |  | 46913 | 91.91 (91.07, 92.76) | -0.89 (-1.53 to -0.51) | |  |
| South | 72885 | 117.03 (116.18, 117.88) |  | 143771 | 136.69 (135.98, 137.41) | 0.41 (0.06 to 0.68) | |  |
| West | 41563 | 118.20 (117.06, 119.34) |  | 77625 | 124.48 (123.60, 125.37) | 0.05 (-0.33 to 0.32) | |  |
| **Race** |  |  |  |  |  |  | |  |
| Black | 31978 | 207.64 (205.33, 209.94) |  | 51312 | 179.32 (177.72, 180.91) | -0.91 (-1.45 to -0.4) | |  |
| Other | 5437 | 110.34 (107.25, 113.42) |  | 19118 | 91.97 (90.65, 93.29) | -0.81 (-1.21 to -0.33) | |  |
| White | 171939 | 109.71 (109.19, 110.23) |  | 268447 | 117.93 (117.48, 118.38) | 0.15 (-0.19 to 0.39) | |  |
| **Sex** |  |  |  |  |  |  | |  |
| Female | 110627 | 104.88 (104.26, 105.50) |  | 147082 | 95.61 (95.12, 96.11) | -0.57 (-0.95 to -0.3) | |  |
| Male | 98727 | 137.72 (136.84, 138.59) |  | 191795 | 155.73 (155.02, 156.45) | 0.35 (0 to 0.67) | |  |
| **Urbanization^e^** |  |  |  |  |  |  | |  |
| Metropolitan | 167014 | 116.46 (115.90, 117.02) |  | 310489 | 138.18 (137.69, 138.67) | 0.46 (-0.08 to 0.78) | |  |
| Nonmetropolitan | 42340 | 126.94 (125.73, 128.15) |  | 76972 | 177.74 (176.45, 179.03) | 1.3 (0.95 to 1.56) | |  |
|  |  |  |  |  |  |  | |  |
| **DMSLD** |  |  |  |  |  |  | |  |
| **Age, y** |  |  |  |  |  |  | |  |
| 25–64 | 79 | 0.07 (0.06, 0.09) |  | 630 | 0.33 (0.30, 0.36) | 6.46 (4.14 to 8.68) | |  |
| ≥65 | 34 | 0.09 (0.06, 0.12) |  | 1713 | 2.92 (2.78, 3.06) | 16.86 (15.13 to 21.48) | |  |
| **Census regions** |  |  |  |  |  |  | |  |
| Midwest^a^ | 28 | 0.07 (0.04, 0.10) |  | 574 | 0.96 (0.88, 1.05) | 13.09 (12.35 to 18.25) | |  |
| Northeast^a^ | 28 | 0.08 (0.05, 0.11) |  | 279 | 0.54 (0.48, 0.61) | 12.19 (10.94 to 14.44) | |  |
| South | 33 | 0.05 (0.04, 0.08) |  | 967 | 0.89 (0.83, 0.95) | 13.01 (11.96 to 15.11) | |  |
| West | 44 | 0.11 (0.08, 0.15) |  | 523 | 0.82 (0.75, 0.90) | 9.48 (8.73 to 10.59) | |  |
| **Race** |  |  |  |  |  |  | |  |
| Black^b^ | 29 | 0.12 (0.07, 0.17) |  | 94 | 0.32 (0.26, 0.40) | 6.03 (3.98 to 10.39) | |  |
| Other^c^ | 43 | 0.28 (0.20, 0.39) |  | 110 | 0.51 (0.41, 0.60) | 8.71 (5.38 to 14.16) | |  |
| White | 89 | 0.05 (0.04, 0.07) |  | 2139 | 0.92 (0.88, 0.96) | 12 (11.16 to 13.08) | |  |
| **Sex** |  |  |  |  |  |  | |  |
| Female | 54 | 0.05 (0.04, 0.07) |  | 1324 | 0.85 (0.80, 0.90) | 12.5 (11.68 to 16.29) | |  |
| Male | 59 | 0.08 (0.06, 0.10) |  | 1019 | 0.79 (0.74, 0.84) | 10.67 (10.01 to 11.48) | |  |
| **Urbanization^e^** |  |  |  |  |  |  | |  |
| Metropolitan | 91 | 0.08 (0.06, 0.10) |  | 1462 | 0.61 (0.58, 0.64) | 10.67 (9.85 to 11.64) | |  |
| Nonmetropolitan | 22 | 0.07 (0.04, 0.11) |  | 440 | 1.01 (0.92, 1.11) | 15.39 (14.24 to 17.47) | |  |
|  |  |  |  |  |  |  | |  |
| **MASLD** |  |  |  |  |  |  | |  |
| **Age, y** |  |  |  |  |  |  | |  |
| 25-64 | 177 | 0.13 (0.11, 0.15) |  | 1189 | 0.63 (0.59, 0.66) | 6.17 (5.47 to 6.91) | |  |
| ≥65 | 50 | 0.14 (0.10, 0.19) |  | 2332 | 4.00 (3.84, 4.17) | 14.75 (14.12 to 15.5) | |  |
| **Census regions** |  |  |  |  |  |  | |  |
| Midwest | 27 | 0.05 (0.03, 0.08) |  | 800 | 1.40 (1.30, 1.50) | 12.81 (12.2 to 15.47) | |  |
| Northeast | 40 | 0.12 (0.09, 0.17) |  | 441 | 0.88 (0.80, 0.97) | 8.55 (7.05 to 10.21) | |  |
| South | 69 | 0.10 (0.08, 0.13) |  | 1458 | 1.38 (1.31, 1.46) | 11.24 (10.44 to 12.45) | |  |
| West | 91 | 0.21 (0.17, 0.26) |  | 822 | 1.34 (1.24, 1.43) | 7.88 (7.14 to 9.08) | |  |
| **Race** |  |  |  |  |  |  | |  |
| Black | 28 | 0.12 (0.08, 0.18) |  | 181 | 0.58 (0.50, 0.67) | 6.45 (4.4 to 8.85) | |  |
| Other^d^ | 20 | 0.17 (0.10, 0.28) |  | 155 | 0.71 (0.60, 0.82) | 10.41 (8.64 to 13.6) | |  |
| White | 193 | 0.12 (0.11, 0.14) |  | 3185 | 1.42 (1.37, 1.48) | 10.32 (9.93 to 10.84) | |  |
| **Sex** |  |  |  |  |  |  | |  |
| Female | 106 | 0.11 (0.09, 0.13) |  | 1928 | 1.31 (1.25, 1.37) | 10.58 (10.02 to 11.29) | |  |
| Male | 121 | 0.14 (0.11, 0.16) |  | 1593 | 1.25 (1.18, 1.31) | 9.38 (8.83 to 10.13) | |  |
| **Urbanization^e^** |  |  |  |  |  |  | |  |
| Metropolitan | 196 | 0.11 (0.09, 0.13) |  | 2238 | 1.01 (0.97, 1.06) | 9.81 (8.97 to 10.58) | |  |
| Nonmetropolitan | 31 | 0.11 (0.08, 0.17) |  | 647 | 1.59 (1.46, 1.72) | 13.38 (11.86 to 15.12) | |  |
| Notes: Superscripts indicate alternative years unavailable/suppressed. a–d replace 1999 with a=2001, b=2011, c=2015, d=2010. e indicates 2020 values replacing 2023. Abbreviations: DMSLD, diabetes mellitus with steatotic liver disease; MASLD, metabolic dysfunction–associated steatotic liver disease; AAMR, age-adjusted mortality rate; AAPC, average annual percent change; CI, confidence interval. | | | | | | | |  |

| **Table S3. Joinpoint-estimated APC and AAPC in SRR1 and SRR2** | | | |
| --- | --- | --- | --- |
| **Characteristics** | **Segment** | **APC (95% CI)** | **AAPC (95% CI)** |
| **SRR1** |  |  |  |
| Overall | 1999-2017 | 11.97 (11.19 to 19.16) | 11.00 (10.44 to 12.54) |
| Overall | 2017-2023 | 8.14 (3.10 to 10.37) |  |
| **Age, y** |  |  |  |
| 25-64 | 1999-2023 | 5.97 (5.28 to 7.06) | 5.97 (5.28 to 7.06) |
| ≥65 | 1999-2007 | 23.85 (12.62 to 104.24) | 16.64 (15.03 to 21.29) |
| ≥65 | 2007-2017 | 15.52 (4.08 to 31.71) |  |
| ≥65 | 2017-2023 | 9.41 (3.68 to 15.59) |  |
| **Census regions** |  |  |  |
| Midwest | 2001-2016 | 15.09 (13.30 to 34.70) | 13.00 (11.87 to 16.66) |
| Midwest | 2016-2023 | 8.64 (2.26 to 11.83) |  |
| Northeast | 2001-2023 | 11.94 (10.91 to 13.77) | 11.94 (10.91 to 13.77) |
| South | 1999-2017 | 13.94 (12.85 to 18.27) | 12.45 (11.76 to 14.33) |
| South | 2017-2023 | 8.11 (3.22 to 10.97) |  |
| West | 1999-2023 | 8.37 (7.76 to 9.38) | 8.37 (7.76 to 9.38) |
| **Race** |  |  |  |
| Black | 2003-2023 | 7.00 (5.11 to 9.86) | 7.00 (5.11 to 9.86) |
| Other | 2011-2023 | 6.92 (4.38 to 10.71) | 6.92 (4.38 to 10.71) |
| White | 1999-2018 | 12.71 (11.97 to 16.65) | 11.57 (11.01 to 13.19) |
| White | 2018-2023 | 7.32 (1.91 to 10.48) |  |
| **Sex** |  |  |  |
| Female | 1999-2016 | 14.51 (13.34 to 17.06) | 12.66 (12.01 to 13.84) |
| Female | 2016-2023 | 8.29 (5.24 to 10.28) |  |
| Male | 1999-2023 | 10.38 (9.76 to 11.37) | 10.38 (9.76 to 11.37) |
| **Urbanization** |  |  |  |
| Metropolitan | 1999-2018 | 11.79 (11.20 to 22.17) | 10.81 (10.24 to 12.8) |
| Metropolitan | 2018-2020 | 1.88 (-3.86 to 10.58) |  |
| Nonmetropolitan | 1999-2020 | 13.81 (12.37 to 16.76) | 13.81 (12.37 to 16.76) |
| **SRR2** |  |  |  |
| Overall | 1999-2017 | 1.78 (1.31 to 5.97) | 1.16 (0.82 to 2.16) |
| Overall | 2017-2023 | -0.66 (-3.77 to 0.76) |  |
| **Age, y** |  |  |  |
| 25-64 | 1999-2023 | 0.59 (-0.05 to 1.57) | 0.59 (-0.05 to 1.57) |
| ≥65 | 1999-2007 | 6.94 (3.27 to 15.92) | 1.70 (1.00 to 3.12) |
| ≥65 | 2007-2023 | -0.82 (-1.36 to -0.24) |  |
| **Census regions** |  |  |  |
| Midwest | 2001-2003 | -21.46 (-32.84 to 1.24) | -1.63 (-2.82 to 0.85) |
| Midwest | 2003-2023 | 0.61 (-1.10 to 3.10) |  |
| Northeast | 2001-2023 | 2.04 (1.17 to 3.52) | 2.04 (1.17 to 3.52) |
| South | 1999-2016 | 2.67 (1.78 to 6.34) | 1.58 (1.04 to 2.69) |
| South | 2016-2023 | -1.02 (-4.46 to 0.62) |  |
| West | 1999-2023 | 1.39 (0.97 to 2.08) | 1.39 (0.97 to 2.08) |
| **Race** |  |  |  |
| Black | 2003-2023 | 0.58 (-0.96 to 3.17) | 0.58 (-0.96 to 3.17) |
| Other | 2011-2021 | -1.81 (-6.61 to 4.36) | -0.40 (-2.11 to 1.69) |
| Other | 2021-2023 | 6.93 (-2.02 to 14.06) |  |
| White | 1999-2018 | 2.16 (1.69 to 4.46) | 1.51 (1.15 to 2.35) |
| White | 2018-2023 | -0.92 (-4.36 to 0.97) |  |
| **Sex** |  |  |  |
| Female | 1999-2023 | 1.11 (0.59 to 2.1) | 1.11 (0.59 to 2.1) |
| Male | 1999-2023 | 1.24 (0.90 to 1.81) | 1.24 (0.90 to 1.81) |
| **Urbanization** |  |  |  |
| Metropolitan | 1999-2020 | 1.41 (0.86 to 2.31) | 1.41 (0.86 to 2.31) |
| Nonmetropolitan | 1999-2020 | 1.51 (0.72 to 2.93) | 1.51 (0.72 to 2.93) |
| **Abbreviations:** APC, annual percent change; AAPC, average annual percent change; SRR1, standardized rate ratio 1 (penetration); SRR2, standardized rate ratio 2 (contribution); AAMR, age-adjusted mortality rate; CI, confidence interval. | | | |

## **Table S4. Sensitivity analyses of DMSLD mortality trends under alternative coding, period, and underlying-cause assumptions**

| **Sensitivity analysis** | **Definition** | **Period** | **Start deaths** | **Start AAMR** | **End deaths** | **End AAMR** | **AAPC (95% CI)** | **Interpretation** |
| --- | --- | --- | --- | --- | --- | --- | --- | --- |
| S1 | E10–E14 AND K76.0 | 1999–2023 | 104 | 0.08 (0.06, 0.09) | 2281 | 0.82 (0.79, 0.85) | 10.77 (9.99 to 11.78) | Directionally consistent with primary DMSLD |
| S2 | E10–E14 AND K75.8 | 1999–2023 | Sparse / suppressed | Not estimated | Sparse / zero | Not estimated | Not estimated | K75.8-only counts were too sparse for reliable Joinpoint modeling |
| S3 | Primary DMSLD: E10–E14 AND K75.8/K76.0 | 1999–2019 | 113 | 0.08 (0.06, 0.09) | 1422 | 0.53 (0.51, 0.56) | 11.16 (10.54 to 12.14) | Increasing trend already evident before pandemic era |
| S4 | E10–E14 AND K76.0 as underlying cause of death | 2000–2023 | 24 | 0.01 (0.00, 0.02) | 940 | 0.31 (0.29, 0.33) | 14.46 (13.46 to 21.51) | Directionally consistent under underlying-cause-only definition, although early counts were low |

**Abbreviations:** DM, diabetes mellitus; DMSLD, diabetes mellitus with steatotic liver disease; MASLD, metabolic dysfunction–associated steatotic liver disease; AAMR, age-adjusted mortality rate; AAPC, average annual percent change; CI, confidence interval. For S4, K76.0 was required to be listed as the underlying cause of death, while diabetes codes E10–E14 were identified anywhere on the death certificate. The start year was 2000 because 1999 estimates were unavailable or suppressed under this restricted definition. Sensitivity analyses should be interpreted as coding-robustness checks rather than clinical validation of proxy-defined MASLD.

**Supplementary figures：**


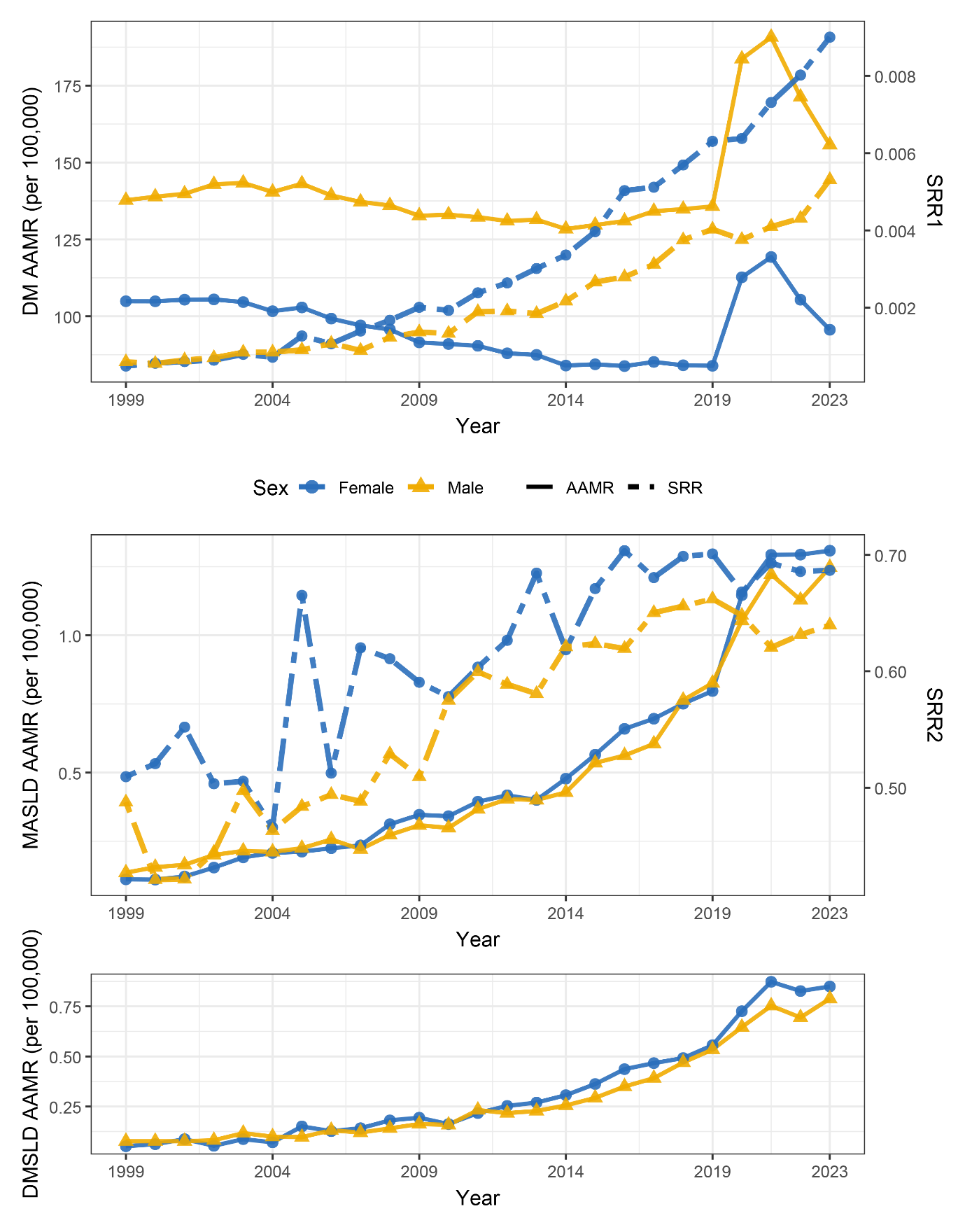


## **Figure S1. Trends in DM, MASLD, and DMSLD AAMR and overlap metrics (SRR1 and SRR2) by sex.**

**Abbreviations:** AAMR, age-adjusted mortality rate; DM, diabetes mellitus; DMSLD, diabetes mellitus with steatotic liver disease; MASLD, metabolic dysfunction–associated steatotic liver disease; SRR, standardized rate ratio; SRR1, penetration (AAMR_DMSLD_/AAMR_DM_); SRR2, contribution (AAMR_DMSLD_/AAMR_MASLD_).


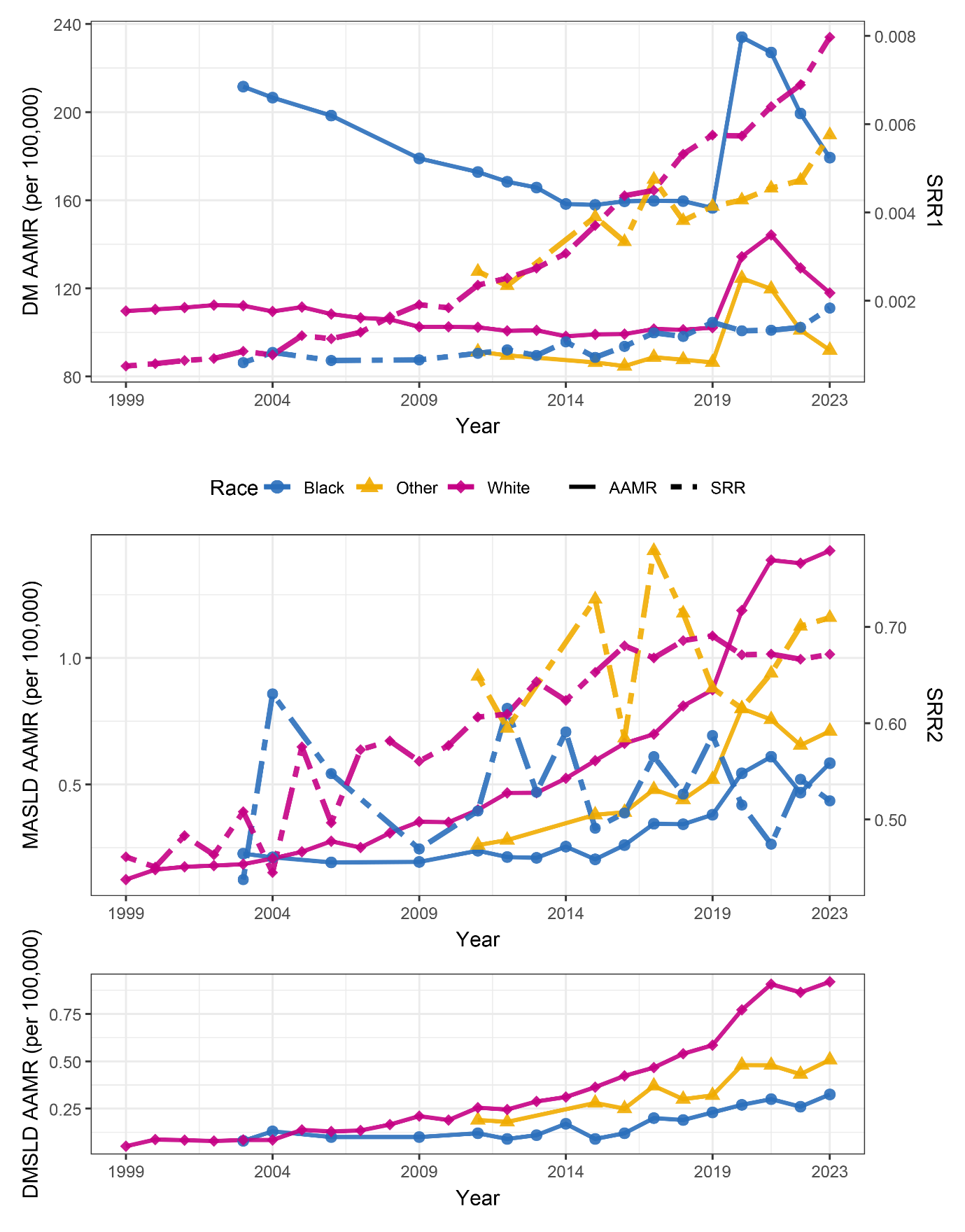


## **Figure S2. Trends in DM, MASLD, and DMSLD AAMR and overlap metrics (SRR1 and SRR2) by race.**

**Abbreviations:** AAMR, age-adjusted mortality rate; DM, diabetes mellitus; DMSLD, diabetes mellitus with steatotic liver disease; MASLD, metabolic dysfunction–associated steatotic liver disease; SRR, standardized rate ratio; SRR1, penetration (AAMR_DMSLD_/AAMR_DM_); SRR2, contribution (AAMR_DMSLD_/AAMR_MASLD_).


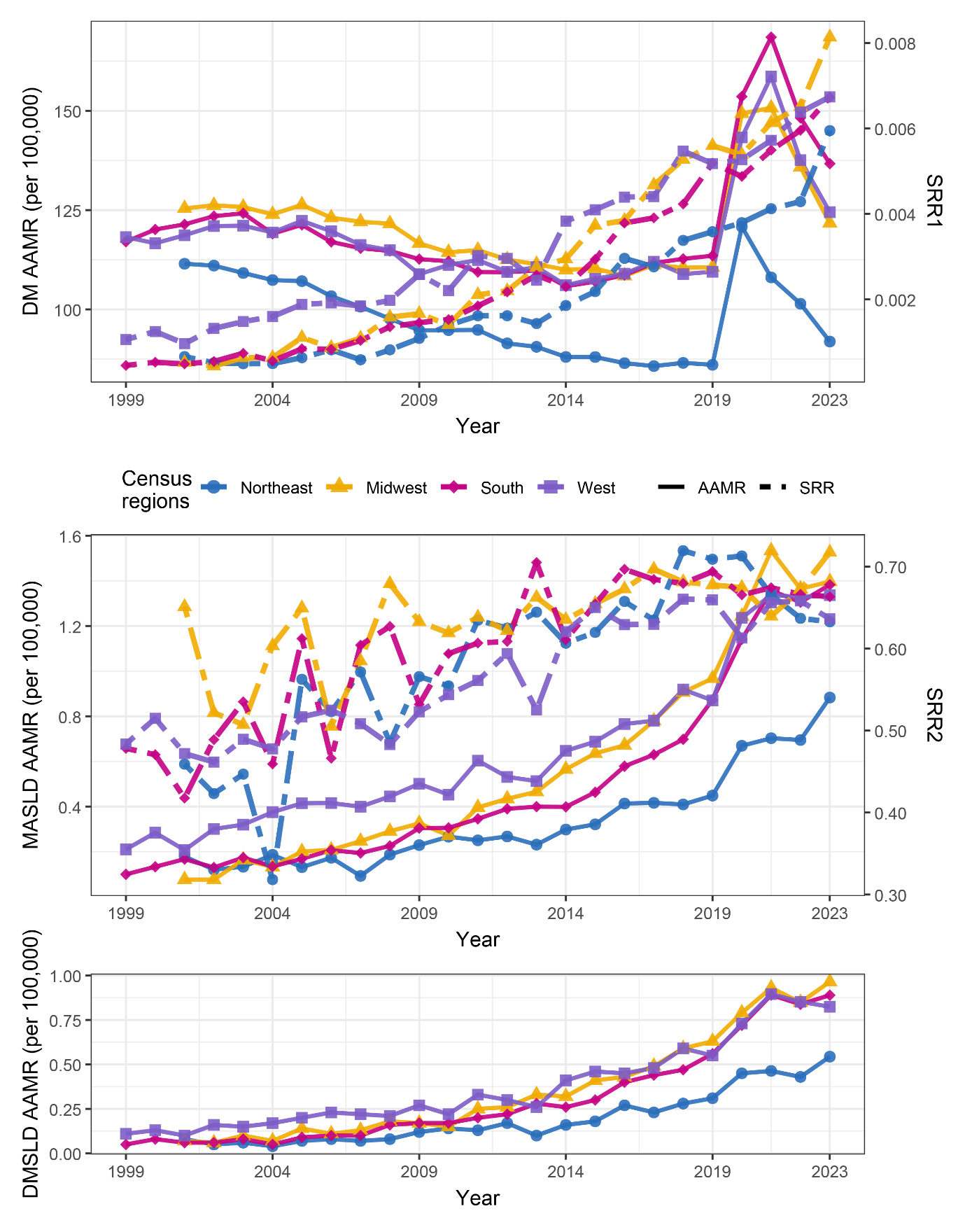


## **Figure S3. Trends in DM, MASLD, and DMSLD AAMR and overlap metrics (SRR1 and SRR2) by census regions.**

**Abbreviations:** AAMR, age-adjusted mortality rate; DM, diabetes mellitus; DMSLD, diabetes mellitus with steatotic liver disease; MASLD, metabolic dysfunction–associated steatotic liver disease; SRR, standardized rate ratio; SRR1, penetration (AAMR_DMSLD_/AAMR_DM_); SRR2, contribution (AAMR_DMSLD_/AAMR_MASLD_).


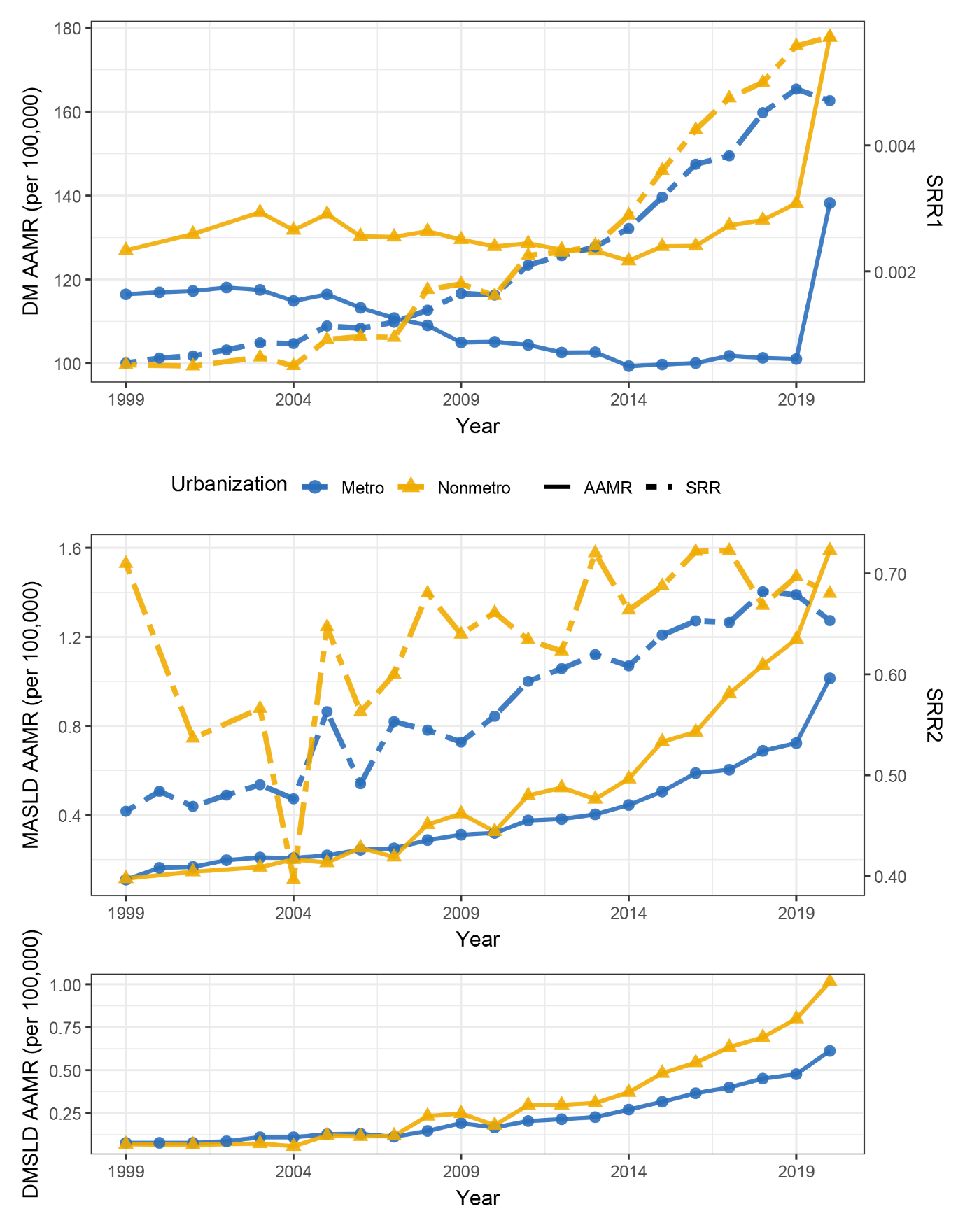


## **Figure S4. Trends in DM, MASLD, and DMSLD AAMR and overlap metrics (SRR1 and SRR2) by urbanization.**

**Abbreviations:** AAMR, age-adjusted mortality rate; DM, diabetes mellitus; DMSLD, diabetes mellitus with steatotic liver disease; MASLD, metabolic dysfunction–associated steatotic liver disease; SRR, standardized rate ratio; SRR1, penetration (AAMR_DMSLD_/AAMR_DM_); SRR2, contribution (AAMR_DMSLD_/AAMR_MASLD_).


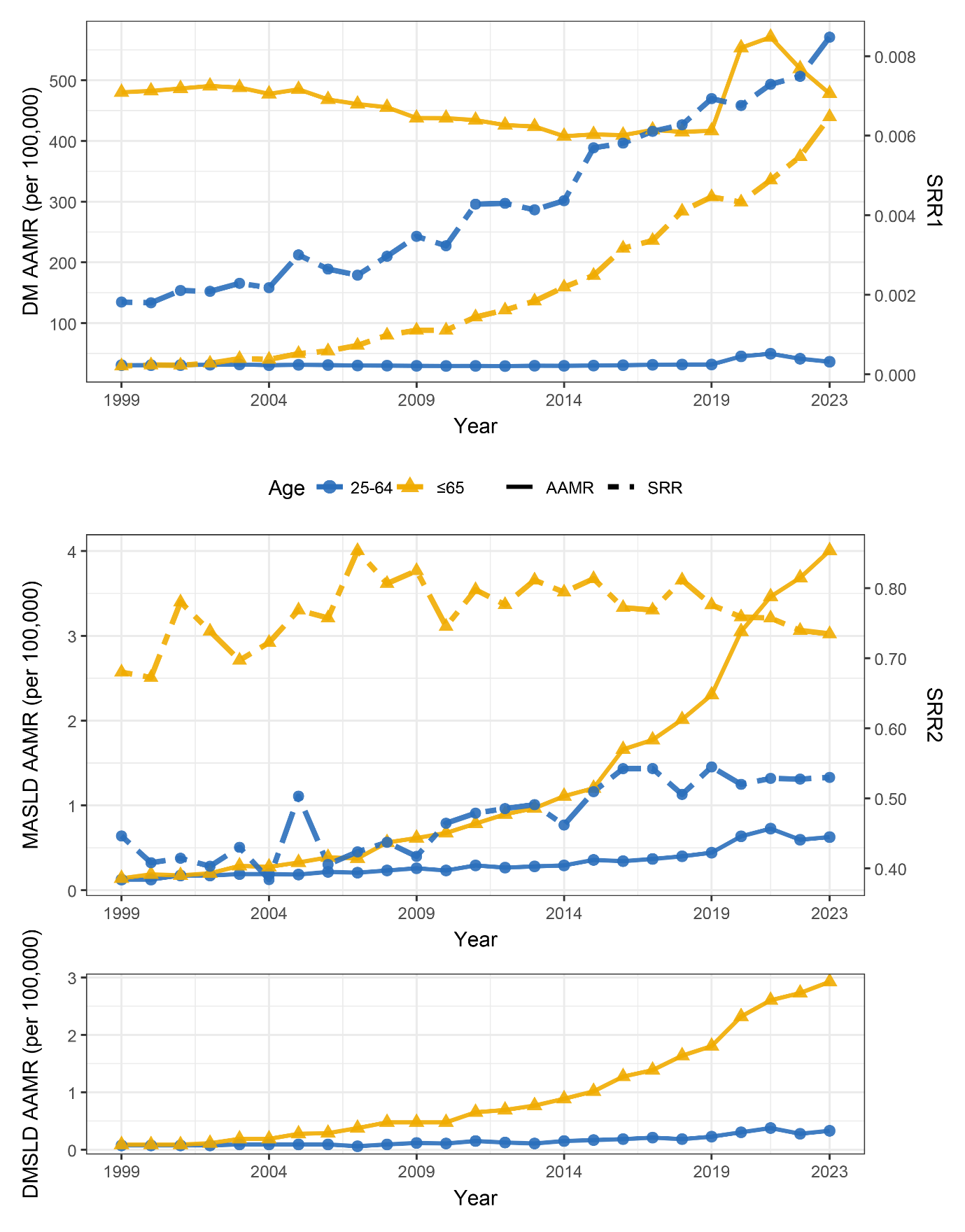


## **Figure S5. Trends in DM, MASLD, and DMSLD AAMR and overlap metrics (SRR1 and SRR2) by age.**

**Abbreviations:** AAMR, age-adjusted mortality rate; DM, diabetes mellitus; DMSLD, diabetes mellitus with steatotic liver disease; MASLD, metabolic dysfunction–associated steatotic liver disease; SRR, standardized rate ratio; SRR1, penetration (AAMR_DMSLD_/AAMR_DM_); SRR2, contribution (AAMR_DMSLD_/AAMR_MASLD_).


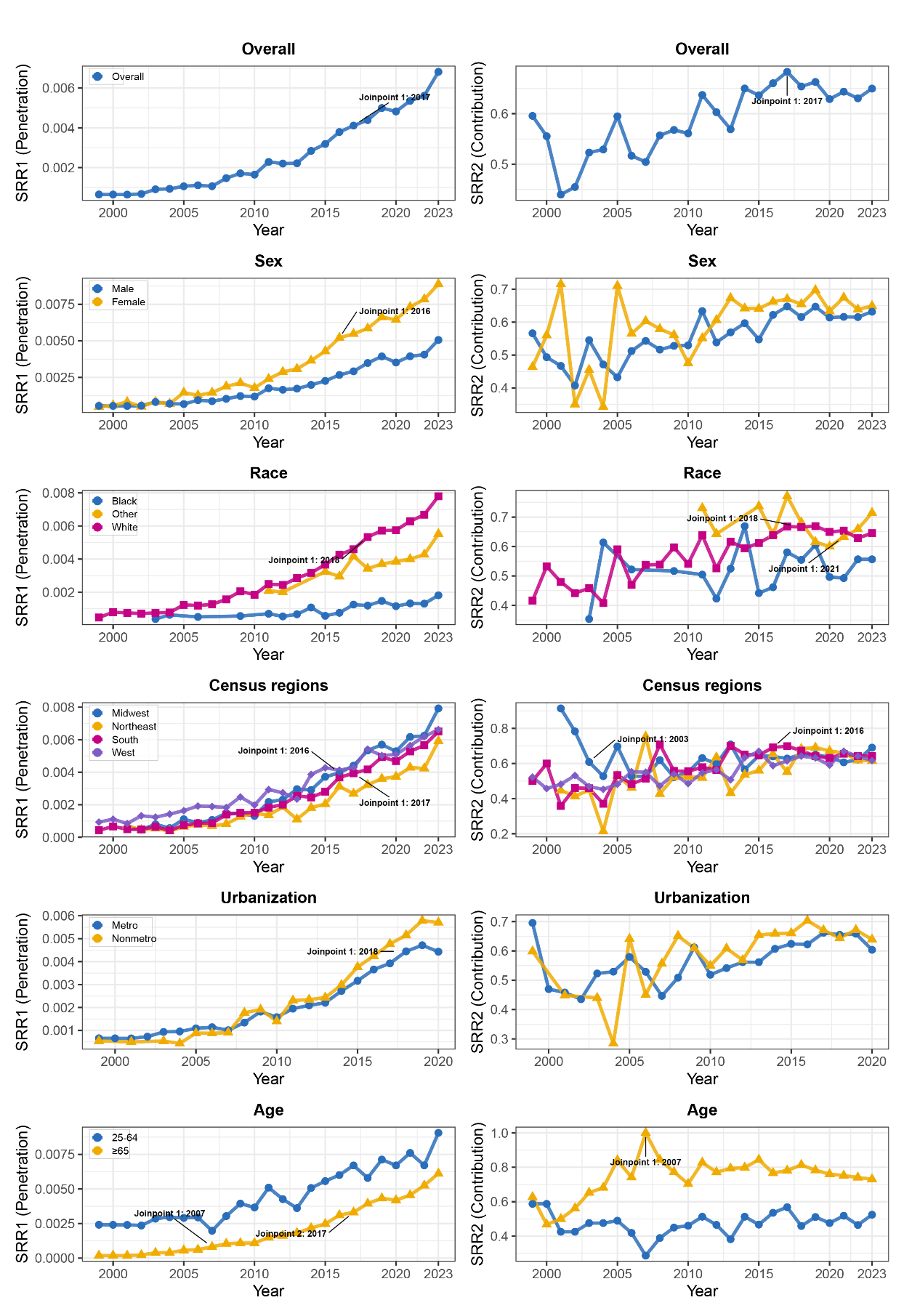


## **Figure S6. Joinpoint-identified trends in SRR1 (penetration) and SRR2 (contribution) by subgroup.**

Abbreviations: SRR, standardized rate ratio; SRR1, penetration (AAMR_DMSLD_/AAMR_DM_); SRR2, contribution (AAMR_DMSLD_/AAMR_MASLD_); AAMR, age-adjusted mortality rate; DM, diabetes mellitus; DMSLD, diabetes mellitus with steatotic liver disease; MASLD, metabolic dysfunction–associated steatotic liver disease.


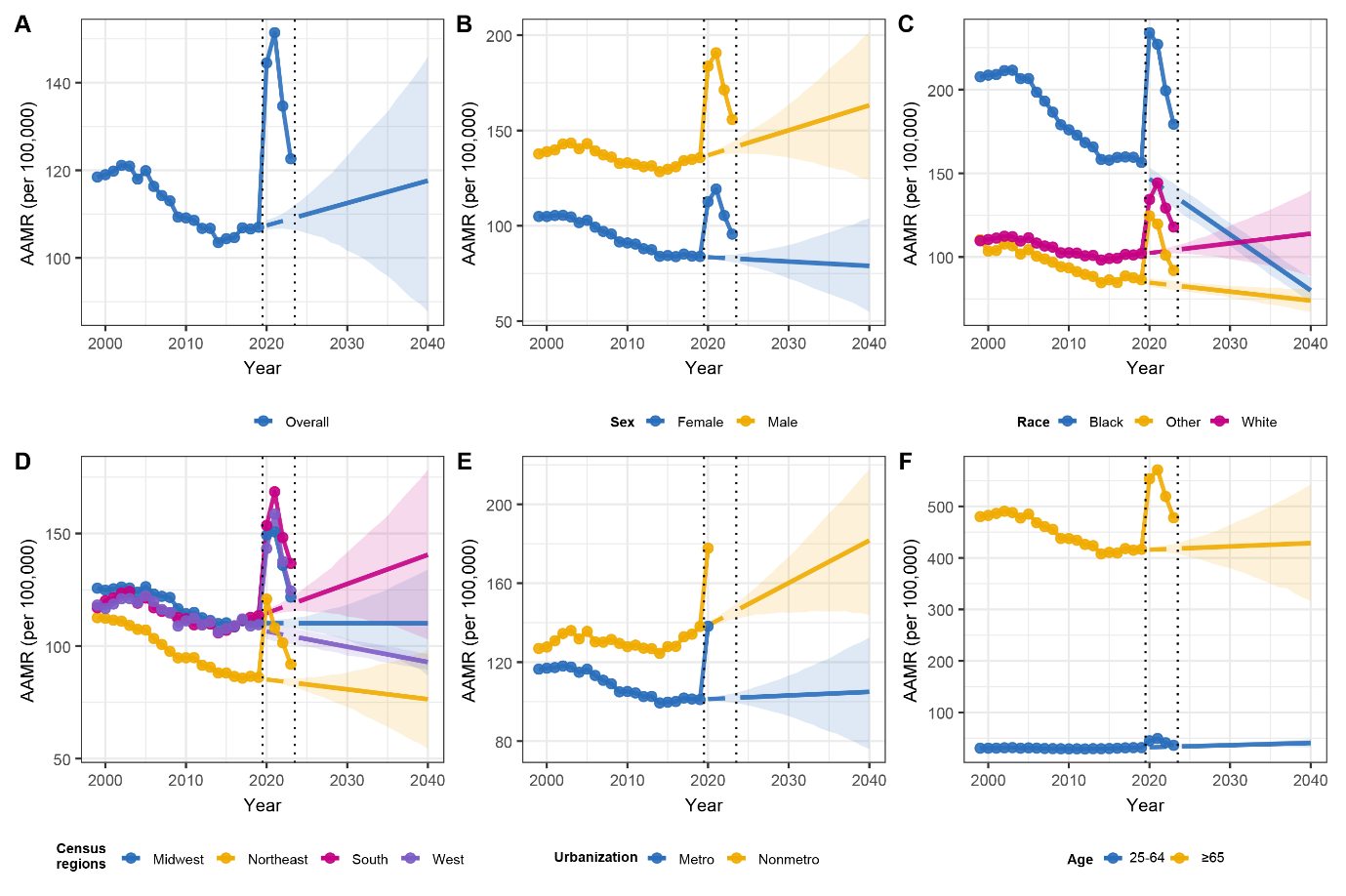


## **Figure S7. Trends in DM AAMR and Prophet-based forecasts through 2040.**

Panels: A, overall; B, sex; C, race; D, census region; E, urbanization; F, age (training: 1999–2019; 2020–2023 excluded).

**Abbreviations:** DM, diabetes mellitus; AAMR, age-adjusted mortality rate.


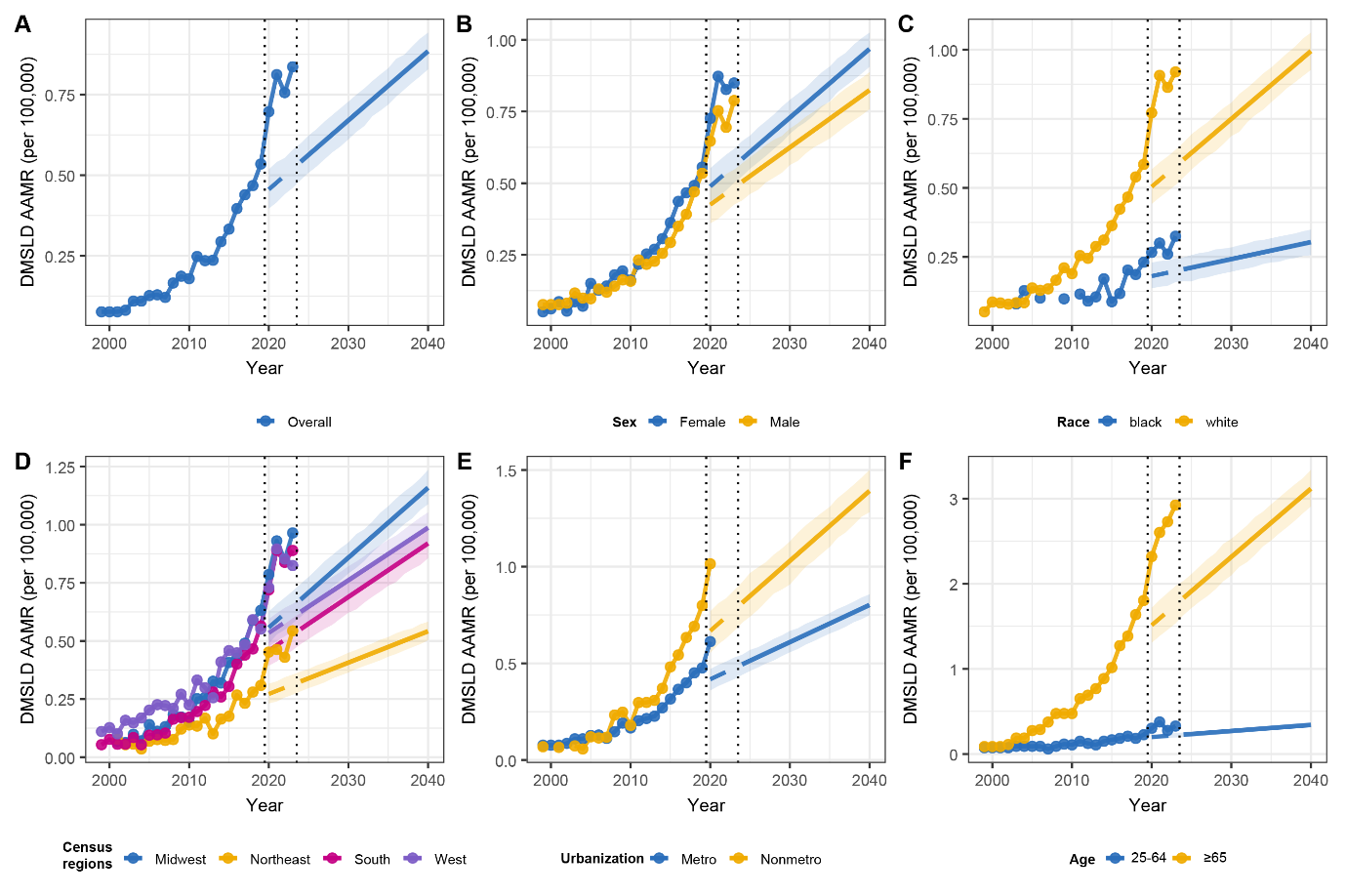


## **Figure S8. Trends in DMSLD AAMR and Prophet-based forecasts through 2040.**

Panels: A, overall; B, sex; C, race; D, census region; E, urbanization; F, age (training: 1999–2019; 2020–2023 excluded).

**Abbreviations:** DMSLD, diabetes mellitus with steatotic liver disease; AAMR, age-adjusted mortality rate.


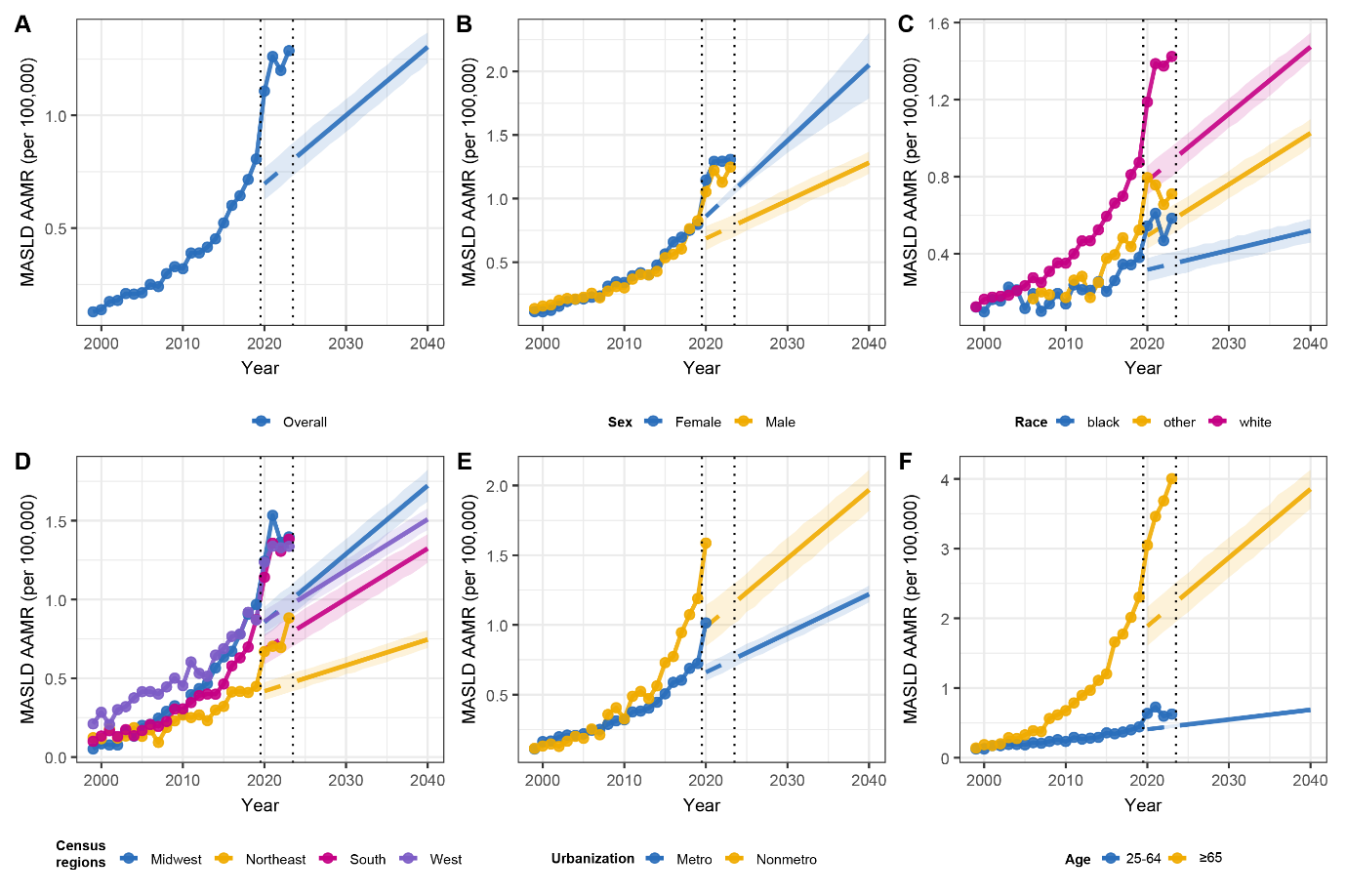


## **Figure S9. Trends in MASLD AAMR and Prophet-based forecasts through 2040.**

Panels: A, overall; B, sex; C, race; D, census region; E, urbanization; F, age (training: 1999–2019; 2020–2023 excluded).

**Abbreviations:** MASLD, metabolic dysfunction–associated steatotic liver disease AAMR, age-adjusted mortality rate.


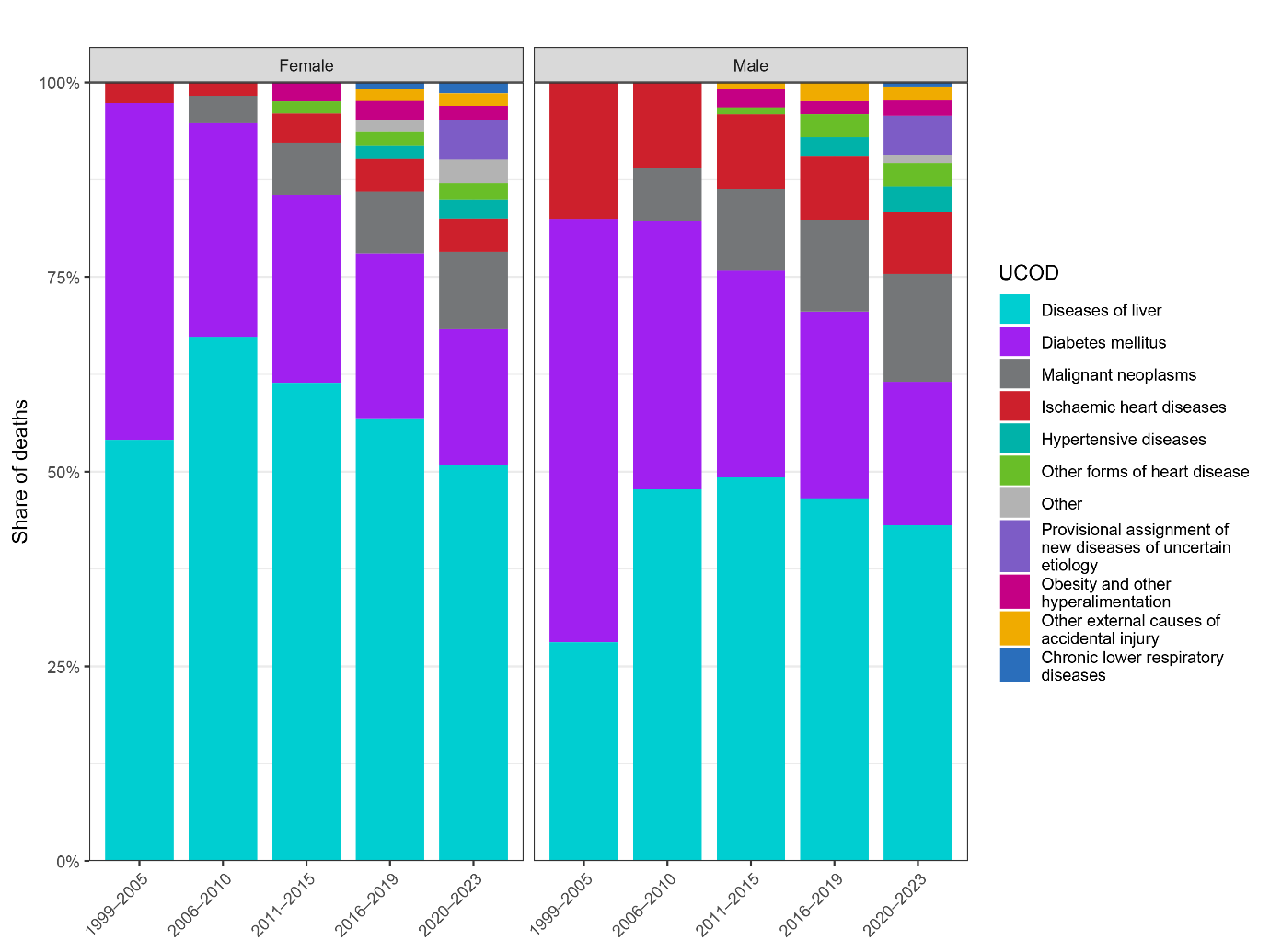


## **Figure S10. UCOD composition among DMSLD overlap deaths (top 10 causes and other) by sex.**

Abbreviations: DMSLD, diabetes mellitus with steatotic liver disease; UCOD, underlying cause of death.


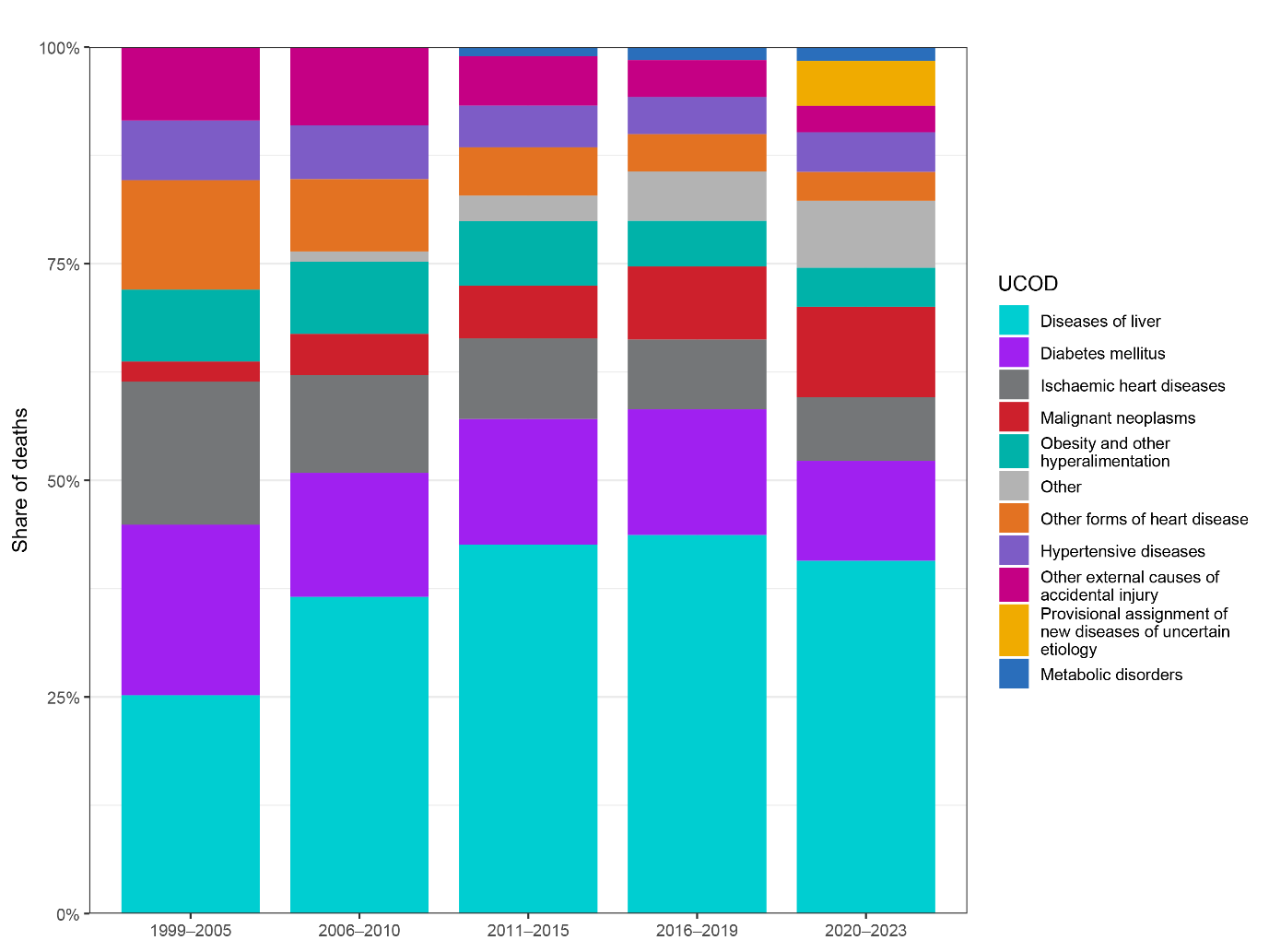


## **Figure S11. UCOD composition among MASLD overlap deaths by period (top 10 causes and other).**

Abbreviations: MASLD, metabolic dysfunction–associated steatotic liver disease; UCOD, underlying cause of death.


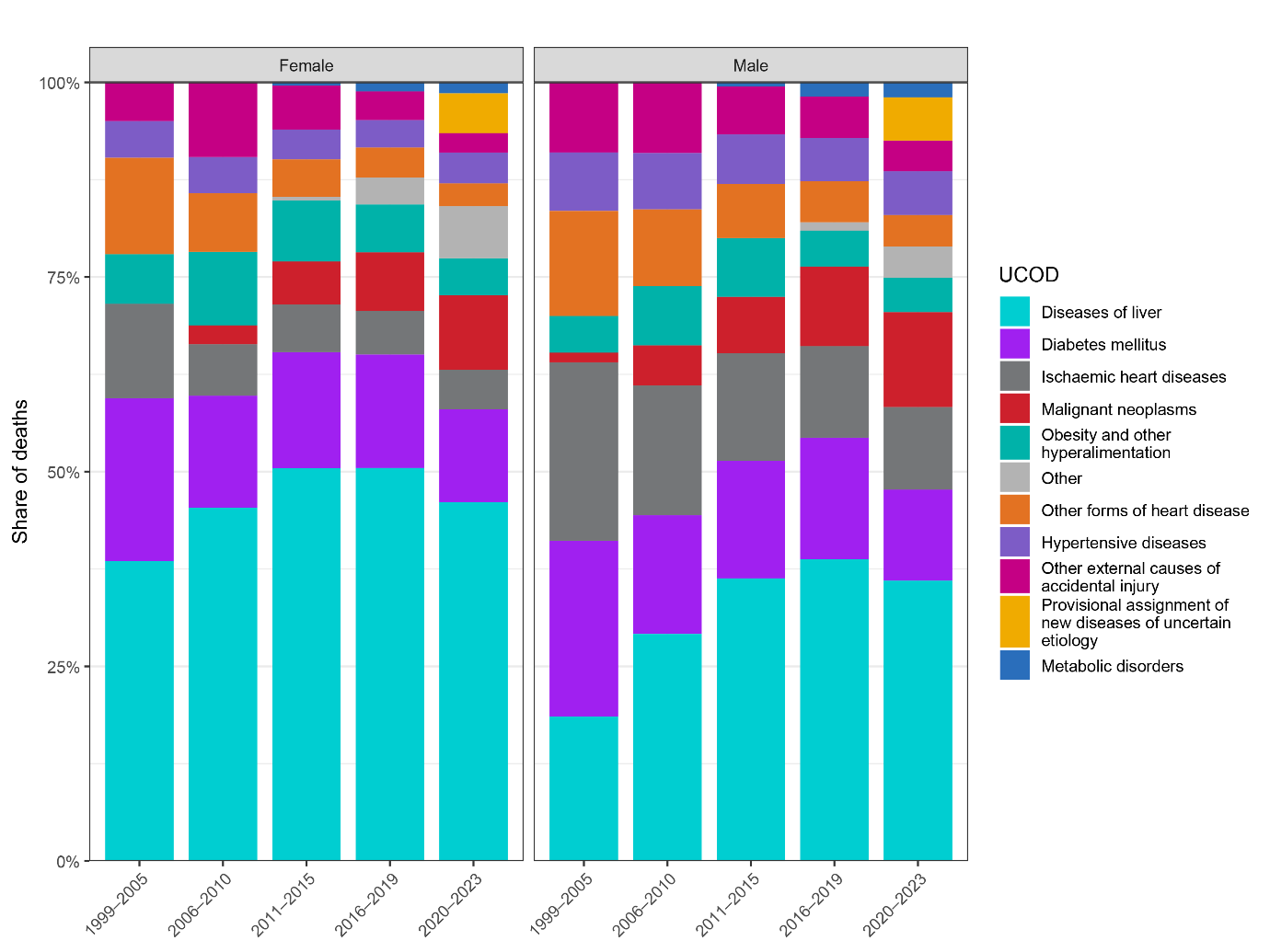


## **Figure S12. UCOD composition among MASLD overlap deaths (top 10 causes and other) by sex.**

Abbreviations: MASLD, metabolic dysfunction–associated steatotic liver disease; UCOD, underlying cause of death.


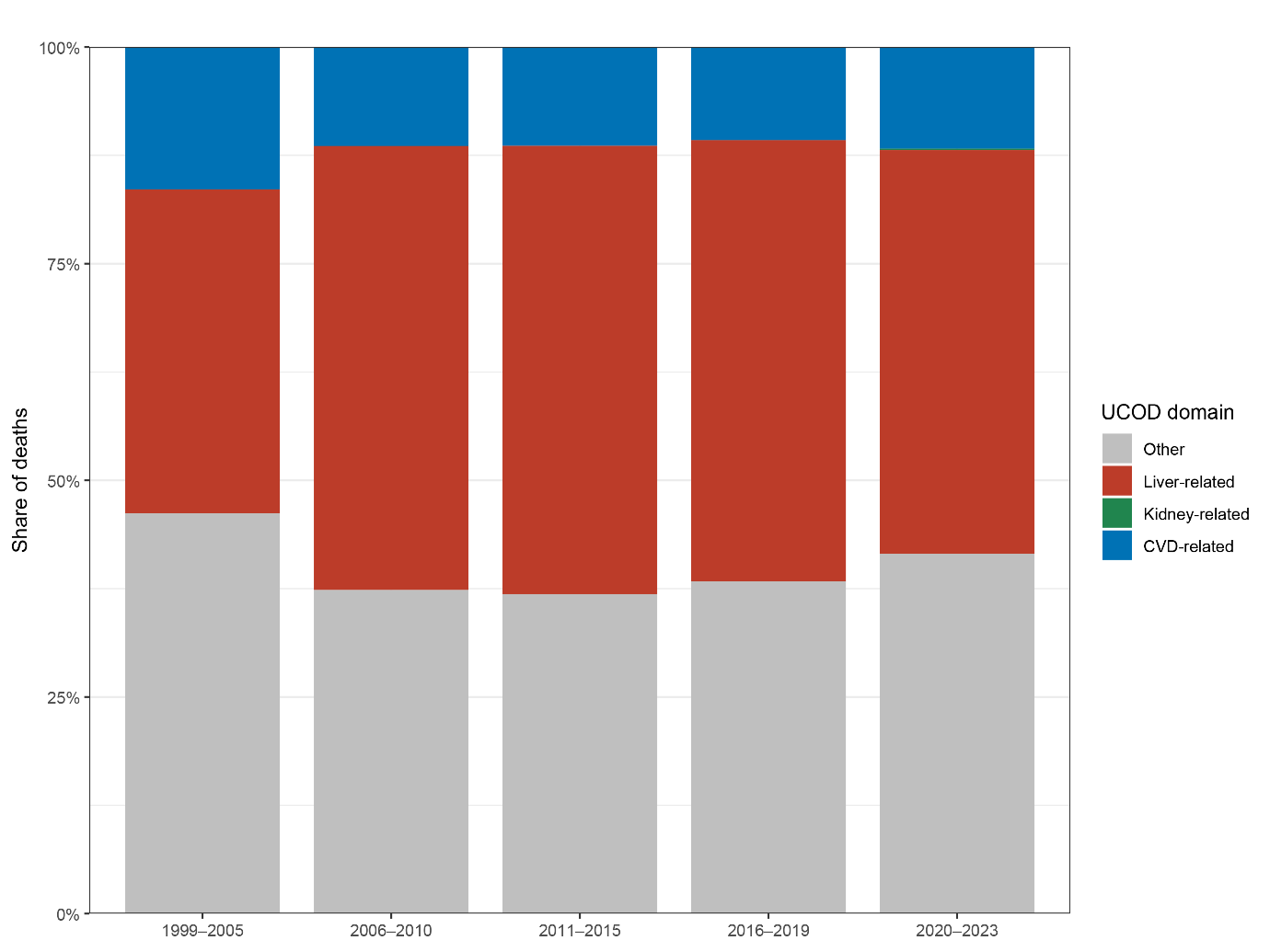


## **Figure S13. UCOD domain composition among DMSLD overlap deaths.**

Abbreviations: DMSLD, diabetes mellitus with steatotic liver disease; UCOD, underlying cause of death; CVD, cardiovascular disease.


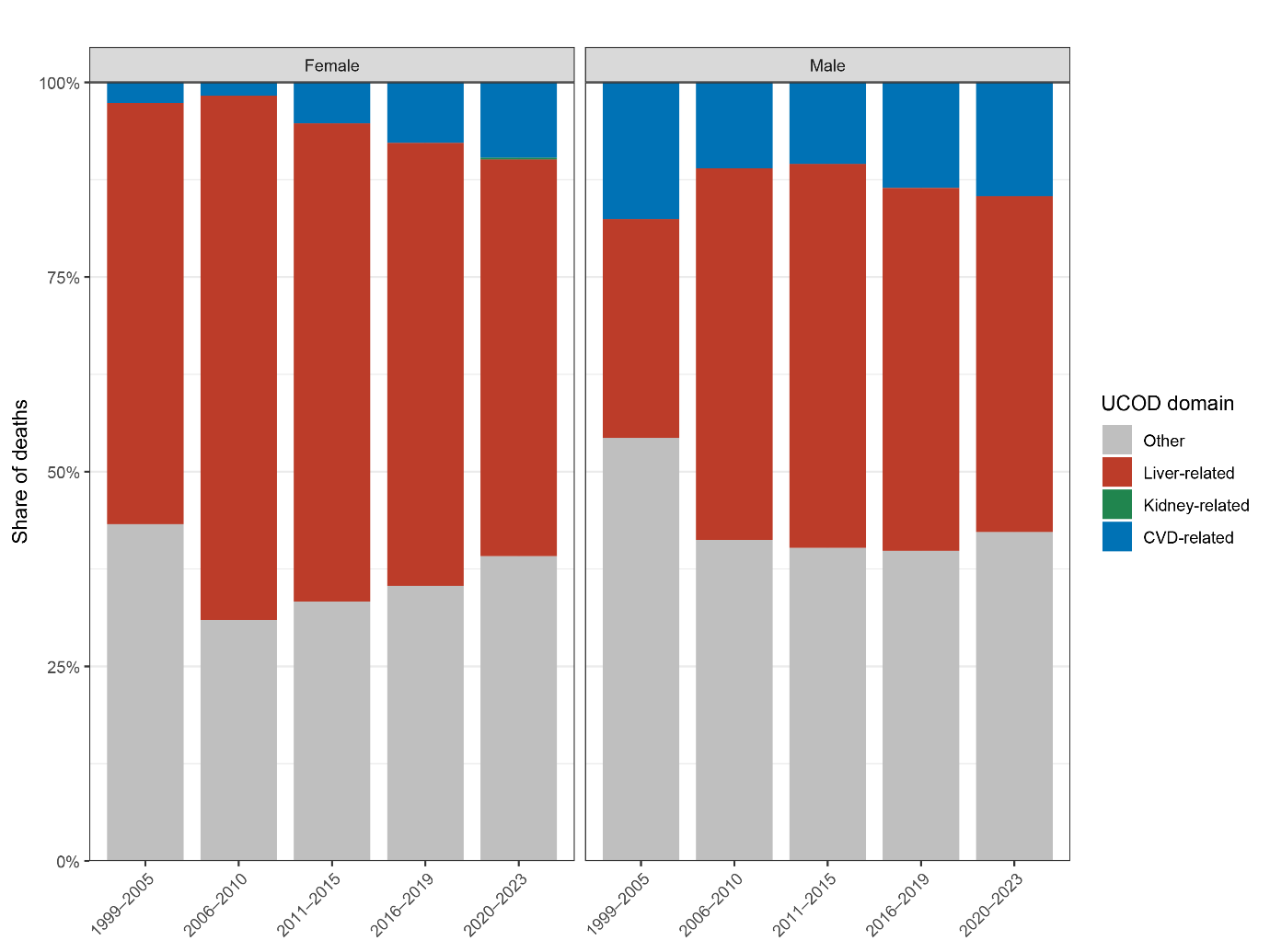


## **Figure S14. UCOD domain composition among DMSLD overlap deaths by sex.**

Abbreviations: DMSLD, diabetes mellitus with steatotic liver disease; UCOD, underlying cause of death; CVD, cardiovascular disease.


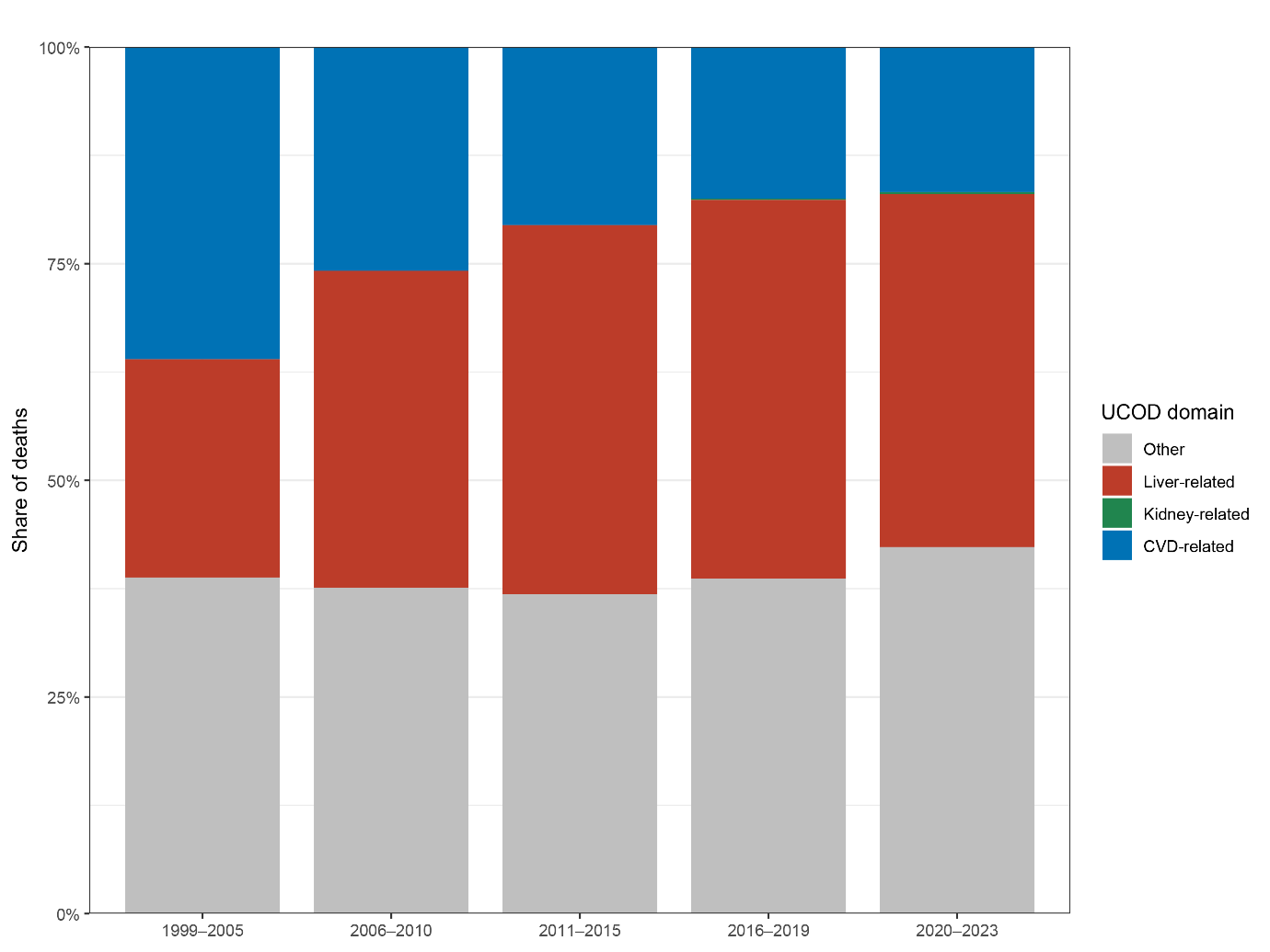


## **Figure S15. UCOD domain composition among MASLD overlap deaths.**

Abbreviations: MASLD, metabolic dysfunction–associated steatotic liver disease; UCOD, underlying cause of death.


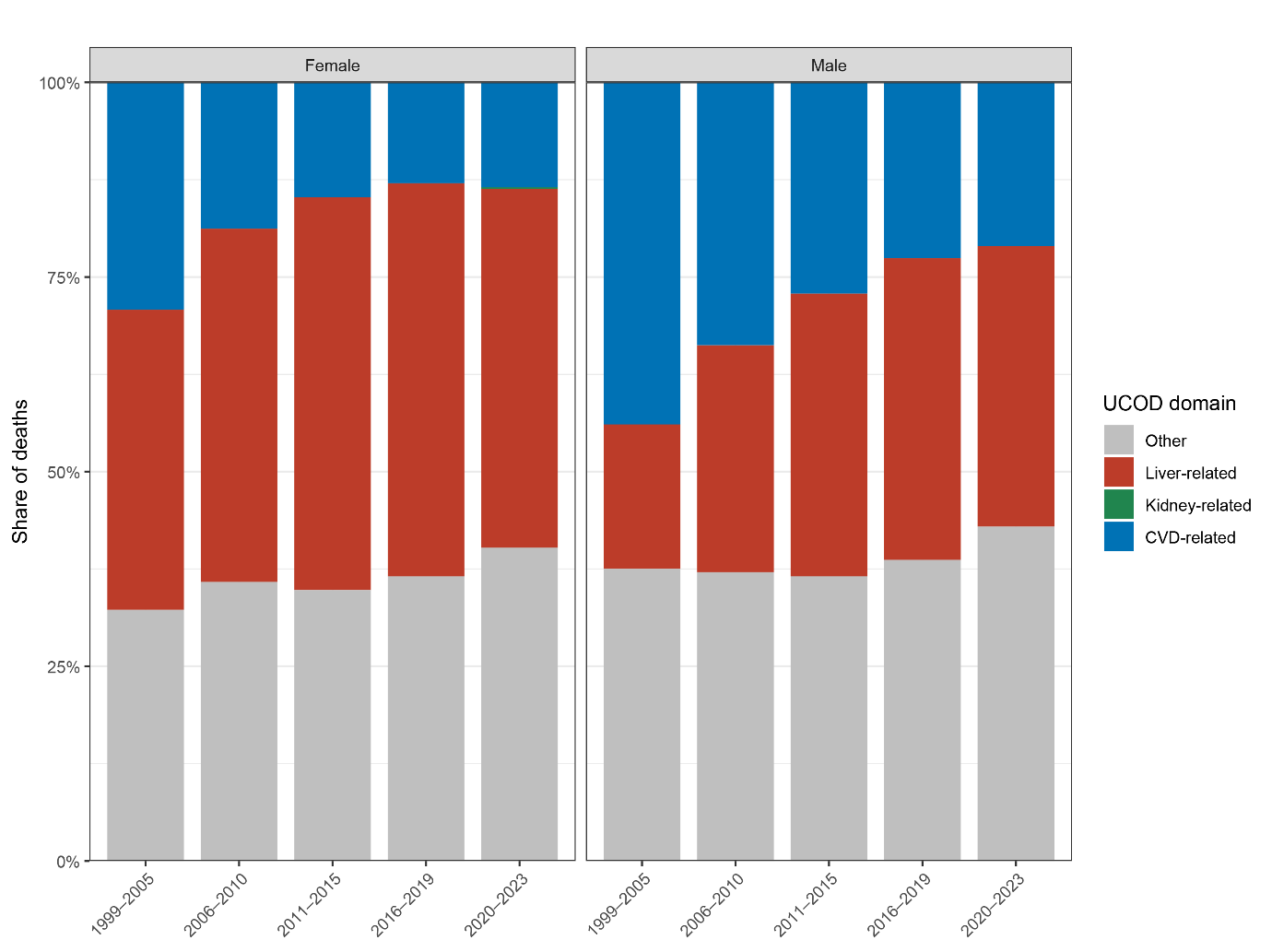


## **Figure S16. UCOD domain composition among MASLD overlap deaths by sex.**

Abbreviations: MASLD, metabolic dysfunction–associated steatotic liver disease; UCOD, underlying cause of death.
